# Targeting mitochondrial metabolism by the mitotoxin bromoxib in leukemia and lymphoma cells

**DOI:** 10.1186/s12964-024-01913-2

**Published:** 2024-11-12

**Authors:** Laura Schmitt, Karina S. Krings, Andre Wolsing, Xabier Buque, Marcel Zimmermann, Hector Flores-Romero, Thomas Lenz, Ilka Lechtenberg, Christoph Peter, Björn Stork, Nicole Teusch, Peter Proksch, Kai Stühler, Ana J. García-Sáez, Andreas S. Reichert, Patricia Aspichueta, Sanil Bhatia, Sebastian Wesselborg

**Affiliations:** 1 Institute for Molecular Medicine I, Medical Faculty, University Hospital Düsseldorf, Heinrich Heine University Düsseldorf, Universitätsstraße 1, 40225 Düsseldorf, Germany; 2 Institute of Biochemistry and Molecular Biology I, Medical Faculty, University Hospital Düsseldorf, Heinrich Heine University Düsseldorf, Universitätsstraße 1, 40225 Düsseldorf, Germany; 3 Department of Physiology, Faculty of Medicine and Nursing, Universidad del País Vasco, Vitoria-gasteiz, Spain; 4 Institute for Genetics, Faculty of Mathematics and Natural Sciences, University of Cologne, 50931 Cologne, Germany; 5 Cologne Excellence Cluster on Cellular Stress Responses in Aging-Associated Diseases (CECAD), University of Cologne, 50931 Cologne, Germany; 6 Ikerbasque, Basque Foundation for Science, Bilbao, 48013 Spain; 7 Achucarro Basque Center for Neuroscience, Leioa, Spain; 8 Molecular Proteomics Laboratory, Biological-Medical-Research Centre (BMFZ), Medical Faculty, University Hospital Düsseldorf, Heinrich Heine University Düsseldorf, Universitätsstraße 1, 40225 Düsseldorf, Germany; 9 Institute of Pharmaceutical Biology and Biotechnology, Faculty of Mathematics and Natural Sciences, Heinrich Heine University Düsseldorf, Universitätsstraße 1, 40225 Düsseldorf, Germany; 10 Biobizkia Health Research Institute, Barakaldo, Spain; 11 Centro de Investigación Biomédica en Red de Enfermedades Hepáticas y Digestivas (CIBERehd), Madrid, Spain; 12 Department of Pediatric Oncology, Hematology and Clinical Immunology, Medical Faculty, University Hospital Düsseldorf, Moorenstraße 5, 40225 Düsseldorf, Germany

**Keywords:** OXPHOS, Metabolism, Protonophore, Leukemia

## Abstract

Targeting mitochondrial metabolism represents a promising approach for cancer treatment. Here, we investigated the mitotoxic potential of the polybrominated diphenyl ether bromoxib, a natural compound isolated from the marine sponge *Dysidea* family. We could show that bromoxib comprised strong cytotoxicity in different leukemia and lymphoma cell lines (such as HL60, HPBALL, Jurkat, K562, KOPTK1, MOLT4, SUPB15 and Ramos), but also in solid tumor cell lines (such as glioblastoma cell lines SJ-GBM2 and TP365MG). Bromoxib activated the mitochondrial death pathway as evidenced by the rapid translocation of Bax to the mitochondria and the subsequent mitochondrial release of Smac. Accordingly, bromoxib-induced apoptosis was blocked in caspase 9 deficient Jurkat cells and Jurkat cells overexpressing the antiapoptotic protein Bcl-2. In addition, we could show that bromoxib functioned as an uncoupler of the electron transport chain with similar rapid kinetics as CCCP in terms of dissipation of the mitochondrial membrane potential (ΔΨm), processing of the dynamin-like GTPase OPA1 and subsequent fragmentation of mitochondria. Beyond that, bromoxib strongly abrogated ATP production via glycolysis as well as oxidative phosphorylation (OXPHOS) by targeting electron transport chain complexes II, III, and V (ATP-synthase) in Ramos lymphoma cells. Thus, bromoxib’s potential to act on both cytosolic glycolysis and mitochondrial respiration renders it a promising agent for the treatment of leukemia and lymphoma.

**Supplementary Information:**

The online version contains supplementary material available at 10.1186/s12964-024-01913-2.

## Introduction

Deregulation of cellular metabolism constitutes a hallmark of cancer as it enables cancer cells to generate essential substrates that promote increased cell division, proliferation, and tumor growth [1]. Hence, targeting mitochondrial metabolism has emerged as a promising approach for surmounting therapy resistance in hematological cancers and solid tumors. Such strategies include the utilization of pharmacological agents aimed at disrupting various components of mitochondrial function, including the electron transport chain (ETC), oxidative phosphorylation (OXPHOS), the tricarboxylic acid (TCA) cycle, or mitochondrial translation [2].

An extensively studied disrupted pathway in cancer involves the activation of Myc (c-Myc), driven by gene amplification, chromosomal translocation, and single-nucleotide polymorphisms [3]. The transcription factor Myc plays a pivotal role in regulating the expression of genes that facilitate anabolic growth. These genes include transporters and proteins engaged in various metabolic processes including fatty acid synthesis, glycolysis, glutaminolysis, serine metabolism, and mitochondrial metabolism [3, 4]. The energy conversion for cellular processes occurs mainly in mitochondria, as the ‘powerhouse’ of the cell [5]. In general, a small proportion of ATP synthesis is accomplished by glycolysis in the cytoplasm, whereas the majority is produced in mitochondria by intimate exchange between the TCA cycle and OXPHOS. Mitochondrial fatty acid β-oxidation (FAO) provides an additional source for ATP generation. The delicate interplay between glycolysis, TCA cycle, and mitochondrial respiration is particularly vulnerable in cancer cells, highlighting the potential of novel therapeutic approaches to target chemotherapy-resistant cancer cells.

**Fig. 1:**
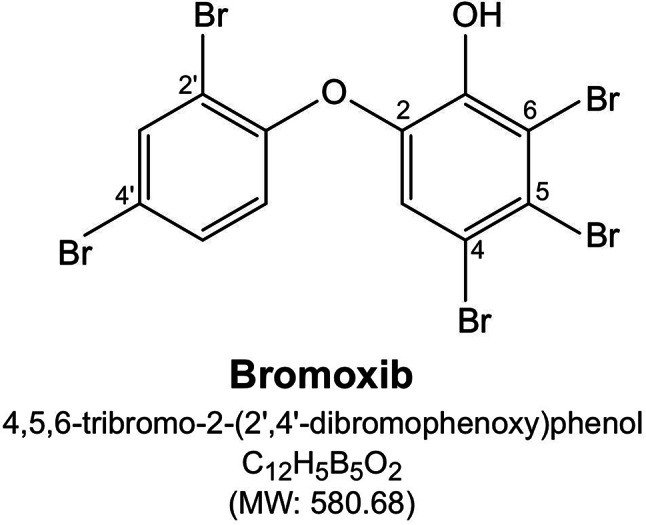
Structure of bromoxib (4,5,6-tribromo-2-(2’,4’-dibromophenoxy)phenol; previously termed P01F08 [6])

We previously showed that the polybrominated diphenyl ether bromoxib (4,5,6-tribromo-2-(2’,4’-dibromophenoxy) phenol; formerly known as P01F08; Fig. 1) displayed a strong cytotoxic potential in different leukemia and lymphoma cell lines (such as Jurkat, THP1 and HL60 and Ramos). In addition, bromoxib showed a 3.2-fold lower IC_50_ value in primary leukemia cells from patients with acute myeloid leukemia (AML) compared to peripheral blood mononucleaar cells (PBMNC) from healthy donors [6, 7]. The natural compound bromoxib was initially isolated from a marine sponge belonging to the *Dysidea* species [8, 9]. The name ‘bromoxib’ is derived from its multiple effects: its composition as a polybrominated diphenyl ether (‘brom’), its role in inhibiting oxidative phosphorylation (‘ox’), and its activity as an inhibitor (‘ib’)” of the ETC.

Here we show that bromoxib exhibited potent cytotoxicity in various leukemia and lymphoma cell lines (including HL60, HPBALL, Jurkat, K562, KOPTK1, MOLT4, SUPB15, and Ramos), as well as in solid tumor cell lines (such as glioblastoma cell lines SJ-GBM2 and TP365MG). Bromoxib activated the intrinsic mitochondrial apoptosis as shown by the translocation of Bax to the mitochondria and the subsequent mitochondrial release of Smac. In addition, bromoxib-induced apoptosis was blocked in Jurkat cells deficient for caspase 9 (as central initiator caspase of the mitochondrial death pathway) and in Jurkat cells overexpressing the antiapoptotic protein Bcl-2. Furthermore, we found that bromoxib acted as an uncoupler of the electron transport chain with similar rapid kinetics as CCCP in terms of disruption of the mitochondrial membrane potential (ΔΨm), processing of the dynamin-like GTPase OPA1 and subsequent mitochondrial fission. In addition, bromoxib induced a rapid release of Ca^2+^ from the endoplasmic reticulum and mitochondria. Interestingly, unlike protonophores such as CCCP, bromoxib strongly abolished ATP production by cytosolic glycolysis and mitochondrial OXPHOS. Inhibition of OXPHOS by bromoxib was mediated by targeting ETC complexes II, III, and the F_1_F_O_ ATP-synthase (complex V) in Ramos lymphoma cells. However, we did not observe any effect of bromoxib on the generation of reactive oxygen species (ROS) and likewise, treatment with the antioxidant *N*-acetylcysteine (NAC) did not affect bromoxib-induced cytotoxicity.

In a mass spectrometric thermal proteome profiling (TPP) approach for potential targets of bromoxib, we identified a protein cluster associated with fatty acid and lipid metabolism, including the enzymes ECH1, ACADVL, ACSL4, and HADHA/B. However, bromoxib did not inhibit fatty acid oxidation (FAO), but rather increased it. This suggests that in a futile attempt to compensate for the bromoxib-induced shutdown of ATP production (due to disruption of the ETC, OXPHOS, and glycolysis), the cells respond by upregulating the metabolic activity of the FAO pathway. In conclusion, the ability of bromoxib to target both glycolysis and mitochondrial respiration renders it as a promising potential anticancer agent.

## Results

### Bromoxib is cytotoxic in leukemia and lymphoma cells and activates the mitochondrial apoptosis pathway

**Fig. 2:**
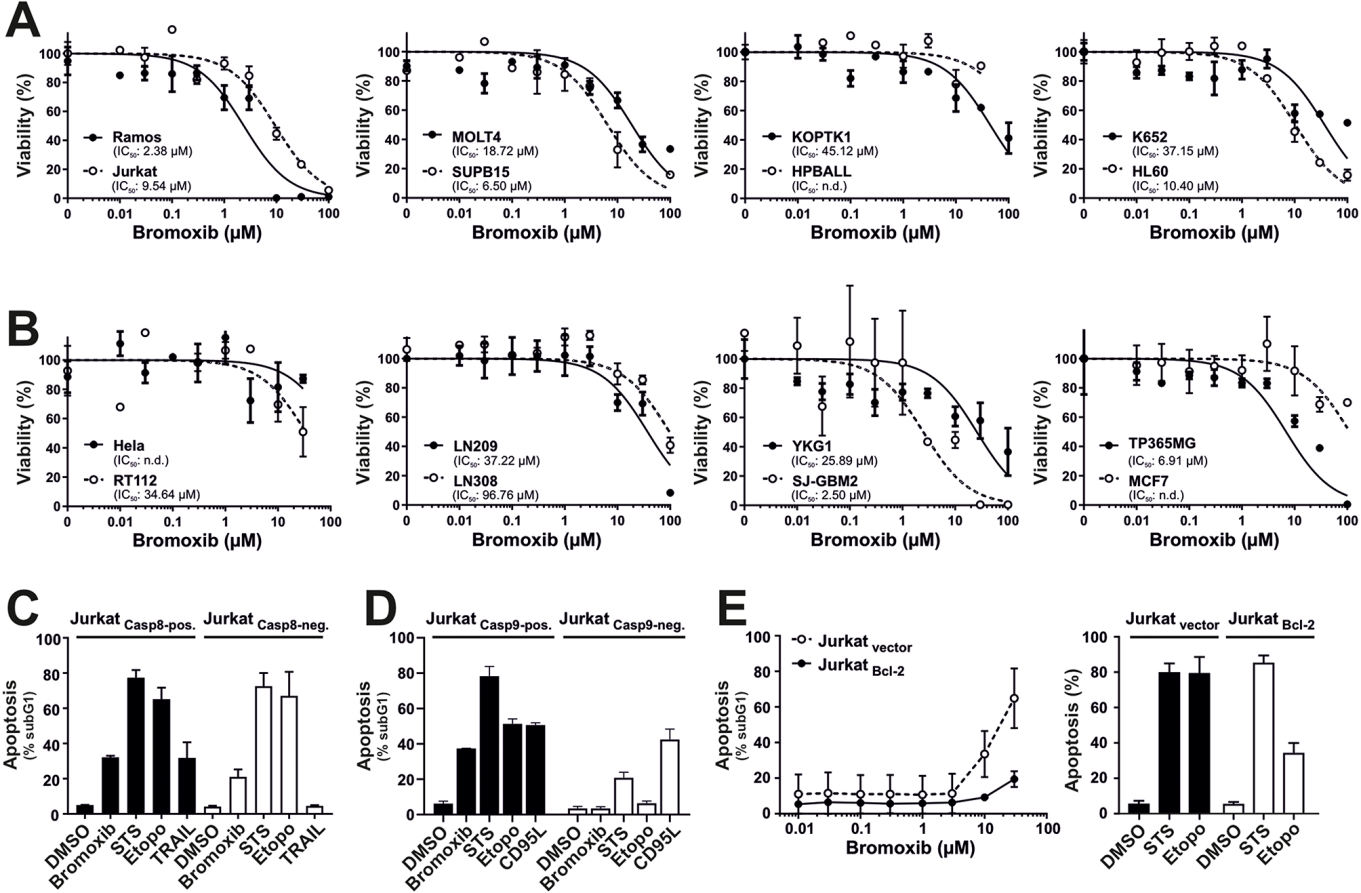
Bromoxib exhibits cytotoxic effects in various cancer cell lines and triggers apoptosis through the intrinsic mitochondria-dependent pathway. Cytotoxicity was determined after 24 h with increasing concentrations of bromoxib in various human cancer cell lines: (**A**) Leukemia and lymphoma cell lines: HL60 (acute myeloid leukemia; AML), HPBALL (T cell acute lymphoblastic leukemia; T-ALL), Jurkat (T-ALL), KOPTK (T-ALL), MOLT4 (T-ALL), SUPB15 (B cell acute lymphoblastic leukemia; B-ALL), K652 (chronic myeloid leukemia; CML), Ramos (Burkitt B cell lymphoma), and (**B**) solid tumor cell lines: HeLa (cervical cancer), MCF7 (breast cancer), RT112 (bladder cancer), LN209, LN308, YKG1, SJ-GBM2, and TP365MG (different glioblastoma cell lines). Cell viability was assessed by AlamarBlue^®^ viability assay. Shown in each graph is the mean ± SD of one representative experiment performed in triplicates. The respective IC_50_ values are shown within the graphs. n.d.=not determinable in the depicted concentration range. (**C**) Bromoxib induces caspase 8 independent and (**D**) caspase 9 dependent apoptosis. After incubating for 24 h, apoptosis-related DNA degradation was observed by measuring propidium iodide-stained apoptotic hypodiploid nuclei using flow cytometry in (**C**) caspase 8-positive and caspase 8-negative Jurkat cells and (**D**) caspase 9-positive and caspase 9-negative Jurkat cells. Cells were stimulated with bromoxib (10 µM), staurosporine (STS; 2.5 µM), etoposide (Etopo; 50 µM), and DMSO (0.1% v/v; diluent control). For stimulation of death receptors in (**C**) TRAIL (Tumor Necrosis Factor Related Apoptosis Inducing Ligand; 40 ng/ml) or (**D**) CD95-ligand (CD95L) (500 ng/ml) were applied. Error bars = mean ± SD of three independent experiments performed in triplicates. (**E**) Bromoxib-induced apoptosis is blocked in the presence of antiapoptotic Bcl-2. Apoptosis induction was analyzed in Jurkat cells stably transfected with vectors encoding Bcl-2 or empty vector. After 24 h apoptosis induction was assessed by propidium iodide staining of apoptotic nuclei and flow-cytometry. Cells were treated with different concentrations of bromoxib (left panel) and respective controls, STS (2.5 µM), Etopo (50 µM), and DMSO (0.1% v/v) (right panel). Error bars = mean ± SD of three independent experiments performed in triplicates

To evaluate the cytotoxic potential, we incubated several hematological cancer cell lines (Fig. 2A) and solid tumor cell lines (Fig. 2B) with the polybrominated diphenyl ether bromoxib for 24 h. Subsequently, cell viability was assessed by AlamarBlue^®^ assay. Thus, bromoxib displayed a high cytotoxicity in Ramos lymphoma cells (IC_50_ 2.38 µM), and leukemia cells, such as Jurkat (IC_50_ 9.54 µM), MOLT4 (IC_50_ 18.72 µM), SUPB15 (IC_50_ 6.50 µM), HL60 (IC_50_ 10.40 µM). It also showed significant cytotoxicity in the glioblastoma cell lines SJ-GBM2 (IC_50_ 2.50 µM) and TP365MG (IC_50_ 6.91 µM), whereas other solid tumor cell lines were less sensitive (Fig. 2A, B).

Next, we investigated which apoptosis signaling pathways were activated by bromoxib. There exist two central apoptosis pathways: The extrinsic death receptor pathway and the intrinsic mitochondrial death pathway. Death receptor-mediated cell death is activated by death receptors (such as CD95/Apo-1/Fas, TRAIL-R1, or TRAIL-R2) upon binding to their respective ligands (e.g., CD95L/Apo-1 L/FasL or TRAIL), leading to the activation of initiator caspase 8, which subsequently activates effector caspase 3. The mitochondrial death pathway is instigated by cellular stress, such as DNA damage induced during radio- and chemotherapy. It commences with the release of cytochrome c from the mitochondria, mediated by proapoptotic Bcl-2 proteins like Bax or Bak. Antiapoptotic Bcl-2 proteins (e.g. Bcl-2, Bcl-xL, Mcl-1) counteract proapoptotic Bcl-2 members by inhibiting the release of mitochondrial cytochrome c and thereby blocking the mitochondrial death pathway [10]. Within the cytosol, cytochrome c binds to the adapter protein Apaf-1, prompting the activation of initiator caspase 9 within a high molecular weight signaling complex known as apoptosome. Upon activation, caspase 9 catalyzes the activation of effector caspases 3 and 7 [11, 12].

In order to study whether bromoxib induces apoptosis via the extrinsic death receptor pathway, we used caspase 8 deficient and proficient Jurkat cells [13] and assessed the apoptosis-related DNA degradation by flow-cytometric measurement of propidium iodide stained apoptotic hypodiploid nuclei using the protocol of Nicoletti et al. [14]. As shown in Fig. 2C, bromoxib induced apoptosis in both, caspase 8 positive and caspas-8 negative Jurkat cells, similar to the broad kinase inhibitor and potent apoptotic stimulus staurosporine (STS) and the DNA-damaging anticancer drug etoposide which are known to induce apoptosis independent of death receptor signaling [15, 16]. As expected, apoptosis-induction via the death receptor ligand TRAIL (tumor necrosis factor-related apoptosis-inducing ligand) was completely blocked in the absence of caspase 8 (Fig. 2C). Hence, the activation of external death receptor signaling could be ruled out in bromoxib-induced apoptosis. However, when we used Jurkat cells deficient for caspase 9 (as the central initiator-caspase of the mitochondrial death pathway) [17], bromoxib-induced apoptosis was completely abrogated (Fig. 2D).

Usually, overexpression of antiapoptotic Bcl-2 proteins (such as Bcl-2, Bcl-xL, or Mcl-1) inhibits the activation of the mitochondrial death pathway [10]. However, since some natural products like viriditoxin can directly activate the mitochondrial apoptosis pathway even in the presence of antiapoptotic Bcl-2 [18], we investigated to what extent bromoxib-induced apoptosis was affected in Bcl-2 overexpressing Jurkat cells. As shown in Fig. 2E, overexpression of Bcl-2 strongly attenuated apoptosis induced by bromoxib and etoposide. As staurosporine is proficient in inducing apoptosis in Bcl-2 or Bcl-xL overexpressing cells [15], apoptosis induction was not affected by Bcl-2.

**Fig. 3:**
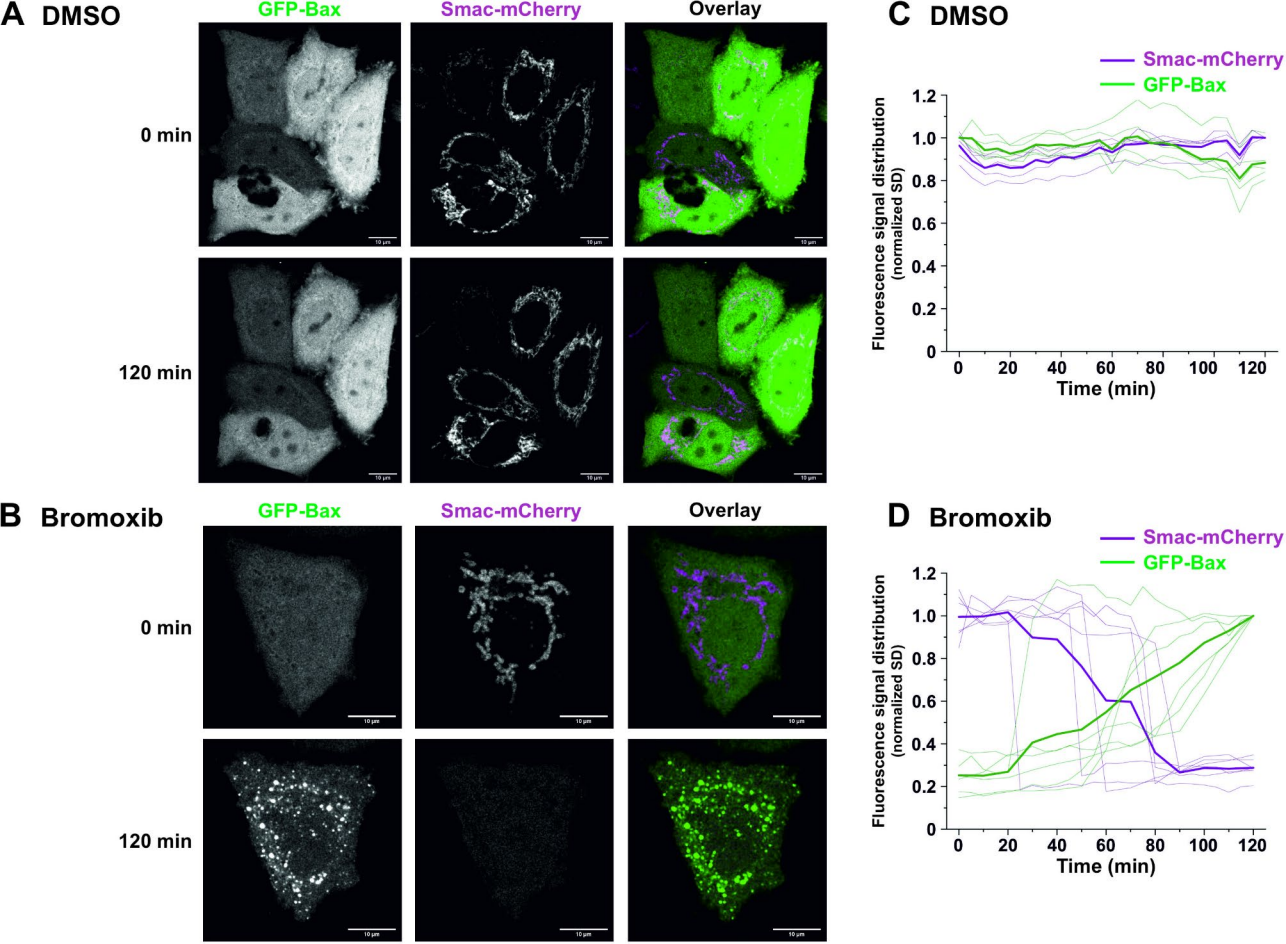
Bromoxib triggers the recruitment of Bax to the mitochondria and the mitochondrial release of the proapoptotic protein Smac. **(A)** and **(B)** confocal live cell imaging of HeLa wild type cells expressing GFP-Bax (green) and Smac-mCherry (magenta) upon treatment with **(A)** DMSO (0.1% v/v,) or **(B)** bromoxib (10 µM). HeLa cells were intentionally used to better track the localization of GFP-Bax and Smac-mCherry. To exclude downstream caspase-mediated effects on cell morphology (such as membrane blebbing), 10 µM of the caspase-inhibitor QVD was added prior to incubation with DMSO or bromoxib. Scale bars indicate 10 μm. **(C)** and **(D)** quantification of the effects of bromoxib on mitochondrial Bax accumulation and Smac release in HeLa wild type cells. The normalized standard deviation (SD) of the fluorescence intensity of GFP-Bax and Smac-mCherry upon treatment with **(C)** DMSO or **(D)** bromoxib was used as a measure of subcellular distribution, in individual cells (*n* = 5–6) from 3 independent experiments. Thinner lines represent measurements of individual cells, while thicker lines represent the average of all recorded cells. A low SD corresponds to homogenous distribution, while a high SD corresponds to an accumulation

Activation of the mitochondrial apoptosis pathway is characterized by the translocation of the proapoptotic Bax protein to the mitochondria, which mediates the subsequent mitochondrial release of proapoptotic factors such as cytochrome c and Smac (also known as Diablo) [10, 12, 19]. To further confirm the involvement of the mitochondrial apoptosis pathway, we monitored the translocation of GFP-Bax to mitochondria and the mitochondrial release of Smac-mCherry in HeLa cells. Thus, we observed that bromoxib induced the mitochondrial translocation of Bax and the release of Smac within 1–2 h (Fig. 3). Taken together, these data indicate, that bromoxib induced apoptosis by activation of the intrinsic mitochondria-dependent signaling pathway.

### Bromoxib causes changes in mitochondrial morphology, resulting in fission that is induced by OMA1-mediated processing of OPA1

**Fig. 4:**
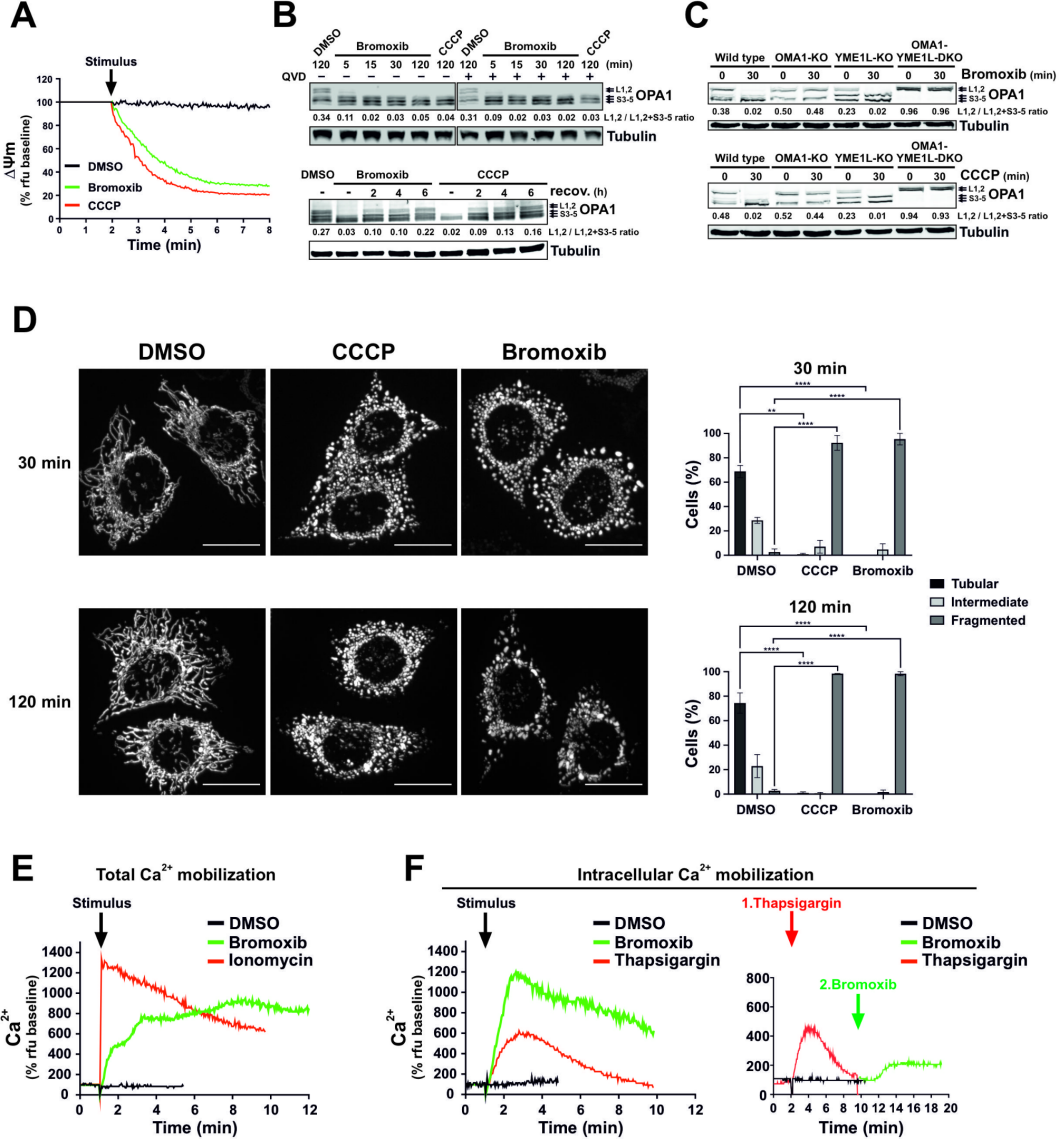
Bromoxib causes a rapid dissipation of the mitochondrial membrane potential (ΔΨm) and changes in mitochondrial morphology, resulting in fission that is regulated by OPA1 and OMA1. **(A)** Within a few minutes of bromoxib treatment, the mitochondrial membrane potential (ΔΨm) decreases rapidly. ΔΨm was monitored in Ramos cells after the addition of DMSO (0.1% v/v), bromoxib (10 µM), or CCCP (protonophore and mitochondrial uncoupler; 10 µM) by flow-cytometry measurement of TMRE fluorescence. **(B) Upper panel**: The kinetics of bromoxib-induced OPA1 cleavage were determined by immunoblotting in Ramos cells treated with 10 µM bromoxib or 10 µM CCCP (as positive control for OPA1 cleavage). Co-treatment with the pan-caspase-inhibitor QVD (10 µM) was conducted to ensure independence from apoptotic signaling. **Lower panel**: To study the recovery of long forms of OPA1, Ramos cells were treated with 10 µM bromoxib or 10 µM CCCP for 30 min, followed by substance removal through centrifugation and a recovery period of up to 6 h. **(C)** The effect of bromoxib (10 µM; upper panel) and of the protonophore CCCP (10 µM; lower panel) on OPA1 processing was determined in murine embryonic fibroblast (MEF) cells deficient for the OPA1 proteases OMA1 (OMA1-KO) or YME1L1 (YMEL-KO) and double knockout of OMA1 and YME1L1 (OMA1-YMEL-DKO). **(B**,** C)** Numbers under OPA1 immunoblots indicate densitometric analyses of the ratio of the long forms of OPA1 (L1 plus L2) to total OPA1 (L1 plus L2 plus S3-5). **(D)** Mitochondrial fission in HeLa cells stably expressing mito-DsRed targeted to the outer mitochondrial membrane after 30 and 120 min of treatment with DMSO (0.1% v/v), bromoxib (10 µM), or CCCP (10 µM). Fragmentation was observed via live cell imaging. Quantification of the mitochondrial morphology was assessed by spinning disc confocal microscopy of the same HeLa cells, categorizing 40 to 60 cells per condition in two independent experiments into tubular, intermediate, or fragmented. The bars show the mean, error bars show the range; statistics: two-way ANOVA with Dunnett’s multiple comparison test (**** = *p* ≤ 0.0001). **(E)** Live measurement of the effect of bromoxib (10 µM) on total Ca^2+^ mobilization in Ramos cells using DMSO (0.1% v/v) as vehicle control and ionomycin (2 µM) as positive control. **(F) Left graph**: Effect of bromoxib on intracellular Ca^2+^ mobilization in Ramos cells. For the measurement of intracellular Ca^2+^ mobilization, cells were washed with Krebs-Ringer buffer containing EGTA as Ca^2+^-chelating agent and treated with bromoxib (10 µM), DMSO (0.1% v/v; diluent control; black line) or thapsigargin (10 µM; positive control). **Right graph**: Live measurement of the effect of thapsigargin (10 µM; red line) followed by bromoxib (10 µM; green line) treatment on intracellular Ca^2+^ mobilization in Ramos cells

Next, we focused on mitochondria as the executioners of the intrinsic apoptosis pathway. Therefore, we assessed the impact of bromoxib on the mitochondrial membrane potential (ΔΨm) in Ramos cells. As depicted in Fig. 4A, bromoxib induced a rapid breakdown of the mitochondrial membrane potential (ΔΨm) within 1–2 min, which was as fast as the protonophore carbonyl cyanide m-chlorophenyl hydrazone (CCCP), which served as a positive control.

In addition to the breakdown of ΔΨm, the mitochondrial death pathway is characterized by mitochondrial fragmentation (fission). Mitochondrial fission is partly regulated by the proteolytic cleavage of the dynamin-like GTPase OPA1 by OMA1 or YME1L1, thereby maintaining the equilibrium between mitochondrial fusion and fission. Consequently, OPA1 serves a pivotal role in mitochondrial homeostasis by sensing mitochondrial stress, such as the breakdown of the ΔΨm, and coordinating mitochondrial quality control and intrinsic apoptosis. Stress-induced cleavage of long isoforms of OPA1 (L-OPA1) and the subsequent formation of short isoforms (S-OPA1) tilts the balance towards mitochondrial fragmentation and increased sensitivity to proapoptotic stimuli [20, 21].

Therefore, we studied the impact of bromoxib on OPA1 processing and observed that bromoxib induced the rapid cleavage of L-OPA1 in Ramos cells within 5 min, which was not affected by caspase-inhibition via QVD (upper immunoblot of Fig. 4B). The recovery of long OPA1 forms upon processing after drug removal serves as an indicator of the recovery of the fusion machinery. To measure L-OPA1 recovery, Ramos cells were treated with bromoxib or CCCP for 30 min, followed by drug removal. Similar to CCCP, the continuous incubation with bromoxib led to L-OPA1 depletion. However, the removal of bromoxib and CCCP allowed the recovery of the long OPA1 forms (Fig. 4B; lower panel), indicating that the effect on mitochondrial fusion is reversible. Usually, S-OPA1 arises through proteolytic cleavage of L-OPA1 by proteases such as OMA1 or YME1L1 [22, 23]. To address which protease (OMA1 or YME1L1) catalyzes the cleavage of L-OPA1 into S-OPA1 forms, mouse embryonic fibroblasts (MEFs) lacking either OMA1, YME1L1, or both were treated with bromoxib or CCCP. The short and long OPA1 forms were then analyzed by immunoblotting (Fig. 4C). The findings indicated that OMA1 is necessary for cleaving L-OPA1 into S-OPA1, as cleavage was absent in OMA1 knockouts. Conversely, YME1L1 knockout did not affect OPA1 cleavage (Fig. 4C). Thus, these data indicate that OMA1 is the responsible protease for bromoxib (and CCCP) induced OPA1 cleavage. In addition, we observed that L-OPA1 depletion coincided with the rapid and pronounced fragmentation of the mitochondrial network within 30 min of treatment of HeLa cells with bromoxib or CCCP (used as positive control for mitochondrial fragmentation; Fig. 4D).

In addition, mitochondrial depolarization associated with a sustained increase in cytosolic Ca^2+^ can also induce mitochondrial fission through activation of the Ca^2+^-dependent phosphatase calcineurin and subsequent dephosphorylation of dynamin-related protein 1 (DRP1) [24, 25]. Therefore, we investigated whether bromoxib was able to induce the mobilization of Ca^2+^ in Ramos cells. As a positive control for Ca^2+^ influx, we used the ionophore ionomycin, which also induces mitochondrial permeability transition opening [26, 27]. Intriguingly, bromoxib induced an increase in cytosolic Ca^2+^ (Fig. 4E) even when extracellular Ca^2+^ was chelated by EGTA (Fig. 4F; left graph). In addition, we used thapsigargin as a sarcoplasmic/endoplasmic reticulum Ca^2+^- ATPase (SERCA) inhibitor to fully deplete the Ca^2+^ stores of the endoplasmic reticulum (ER). To determine the origin of the released Ca^2+^, thapsigargin was initially applied to release all Ca^2+^ stored within the ER. However, subsequent application of bromoxib was still able to mediate a residual release of intracellular Ca^2+^ (Fig. 4F; right graph), indicating that the Ca^2+^ mobilized by bromoxib originates from the ER and additionally from the mitochondria.

As severe mitochondrial damage and oxidative stress are often interdependent, we investigated the effect of bromoxib on cellular reactive oxygen species (ROS) generation. However, measurements with the fluorescent dye H_2_-DCF-DA revealed no increase in ROS production (Suppl. Figure 1 A,B). Accordingly, treatment with the antioxidant *N*-acetylcysteine (NAC) did not affect bromoxib-induced toxicity (Suppl. Figure 1 C).

### Bromoxib inhibits glycolysis and mitochondrial metabolism

**Fig. 5:**
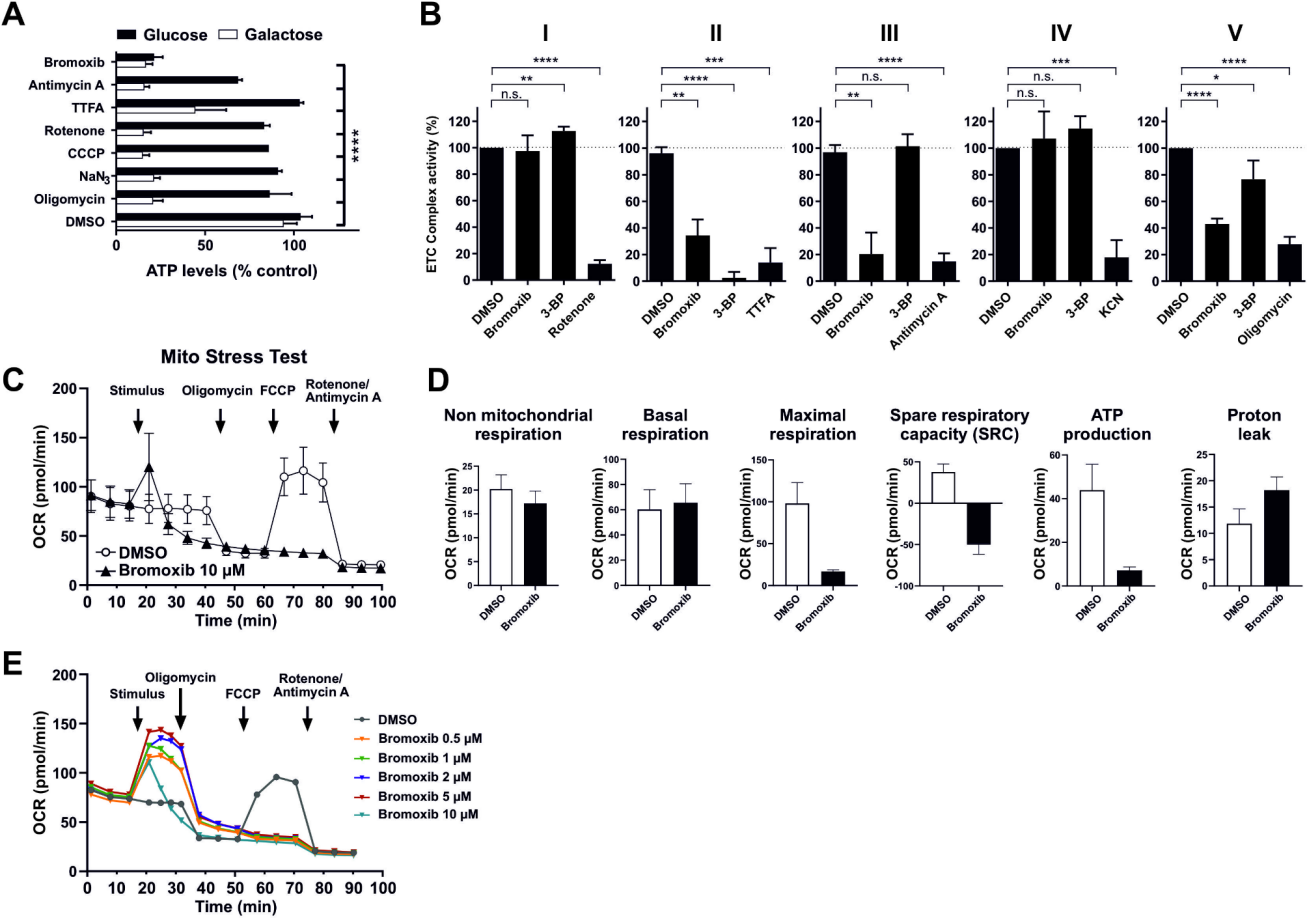
Bromoxib specifically targets mitochondrial metabolism, leading to the inhibition of both OXPHOS and glycolysis. **(A)** The impact of bromoxib (10 µM) and several known mitotoxins on the ATP levels in Ramos cells. The cells were treated with the specified agents in a complete growth medium containing either glucose or galactose as the sole available sugar. The following complex-specific inhibitors of the ETC were used: complex I: 10 µM rotenone; complex II: 10 µM thenoyltrifluoroacetone (TTFA); complex III: 10 µM antimycin A; complex IV: 1 mM sodium azide (NaN_3_); complex V: 10 µM oligomycin; mitochondrial uncoupler and protonophore: 1 µM CCCP and 10 µM bromoxib as well as DMSO (0.1% v/v) as vehicle control. ATP-levels were measured using the luminescence-based mitochondrial ToxGloTM assay (Promega). The depicted values were normalized to cells treated with DMSO in glucose containing growth medium (set to 100%). Error bars = mean ± SD of three independent experiments performed in triplicates; p-values were calculated by two-way ANOVA with the Holm-Sidak post-test; (**** = *p* ≤ 0.0001). **(B)** Bromoxib inhibits ETC complexes II, III, and ATP-synthase (complex V). The activities of the individual complexes of the respiratory chain were measured after treatment with bromoxib (10 µM) or the respective complex inhibitors (complex I: 10 µM rotenone; complex II: 10 mM thenoyltrifluoroacetone (TTFA) or 10 µM 3-bromopyruvate (3-BP); complex III: 10 µM antimycin A; complex IV: 1 mM potassium cyanide (KCN); complex V: 10 µM oligomycin) for 15 min using the MitoCheck^®^ kit (Cayman Chemical; utilizing mitochondria isolated from bovine heart). Depicted activities were normalized to cells treated with DMSO (0.1% v/v). Statistical analysis: Unpaired t test; two-tailed (**** = *p* ≤ 0.0001). **(C)** The effect of bromoxib on the mitochondrial oxygen consumption rate (OCR; pmol O_2_/min) was determined in a Seahorse XFe96 Extracellular Flux Analyzer with the Mito Stress Test Kit. HeLa cells (15000 cells per well) were subjected to acute injection with bromoxib (10 µM) or DMSO (0.1% v/v) as a solvent control, and the oxygen consumption rate was monitored over a 100-min duration. Error bars = mean ± SD of three independent experiments. The following complex-specific inhibitors of the ETC were used: complex I: 0.5 µM rotenone; complex III: 0.5 µM antimycin A; complex V: 1 µM oligomycin; mitochondrial uncoupler and protonophore: 1 µM FCCP. **(D)** Various mitochondrial respiration parameters obtained from the Mito Stress Test were compared in HeLa cells treated with bromoxib (10 µM) and DMSO (0.1% v/v). Error bars = mean ± SD of three independent experiments. **(E)** Effect of different bromoxib concentrations on OCR. For the Mito Stress Test HeLa cells (15000 cells per well) were acutely treated with various concentrations of bromoxib (ranging from 0.5 µM to 10 µM) or DMSO (0.1% v/v) as the solvent control. The OCR) was then monitored over a period of 90 min. The following complex-specific inhibitors of the ETC were used: complex I: 0.5 µM rotenone; complex III: 0.5 µM antimycin A; complex V: 1 µM oligomycin; mitochondrial uncoupler and protonophore: 1 µM FCCP

Given that one of the primary functions of mitochondria is the supply of cellular ATP, we examined the extent to which bromoxib affects cellular ATP levels. To distinguish the effects on glycolysis from OXPHOS, cells were provided with either glucose or galactose as the sole sugar source within the medium. The glycolytic consumption of galactose instead of glucose does not result in a net ATP gain, thereby forcing the cell to rely exclusively on OXPHOS for ATP production. This renders the cell particularly susceptible to inhibitors of the ETC [28]. As shown in Fig. 5A, bromoxib induced a marked reduction in ATP levels under both glucose and galactose conditions, which was distinct from all other ETC inhibitors tested. To investigate whether bromoxib could directly inhibit one of the five electron ETC complexes, we analyzed the activity of each complex in purified mitochondria following bromoxib treatment. Intriguingly, bromoxib exhibited marked inhibitory activity and selectively targeted ETC complexes II, III, and the ATP-synthase (complex V) (Fig. 5B).

To confirm the role of bromoxib as an inhibitor of mitochondrial respiration, HeLa cells were treated with bromoxib and mitochondrial respiration was monitored by oxygen consumption rate (OCR) using the Agilent Seahorse system with the Mito Stress Test protocol. As shown in Fig. 5C, treatment with 10 µM bromoxib immediately caused a transient increase in OCR before mitochondrial respiration dropped to almost zero. Interestingly, subsequent addition of FCCP did not result in an increase in OCR, consistent with the observation that bromoxib impaired ETC complexes II and III. Subsequently, the non-mitochondrial respiration, basal respiration, maximal respiration, spare respiratory capacity (SRC), ATP production and proton leak were calculated from the OCR measurements (Fig. 5D). Thus, bromoxib induced a response and triggered a pronounced reduction of maximal respiration as well as spare respiratory capacity (Fig. 5D). In addition, bromoxib provoked a proton leak and completely abrogated the ATP production (Fig. 5A, D). These findings support the notion that bromoxib, like CCCP, might function as an uncoupler and in addition blocks OXPHOS complexes, thereby impairing mitochondrial morphology and respiratory metabolism.

### Bromoxib acts as a mitochondrial uncoupler

In general, a protonophore can be defined as a proton translocator or ionophore that facilitates proton transfer across membranes. Prominent examples of protonophores are compounds like CCCP, 2,4-dinitrophenol, or triphenylphosphonium, which are known for their cytotoxic potential in various cancer cell types, including breast cancer, lung cancer, neuroblastoma, and glioblastoma cells [29–31]. These agents function by disrupting the normal proton gradient across the inner mitochondrial membrane, thereby uncoupling OXPHOS which results in increased oxygen consumption rates, yet diminished ATP production.

**Fig. 6:**
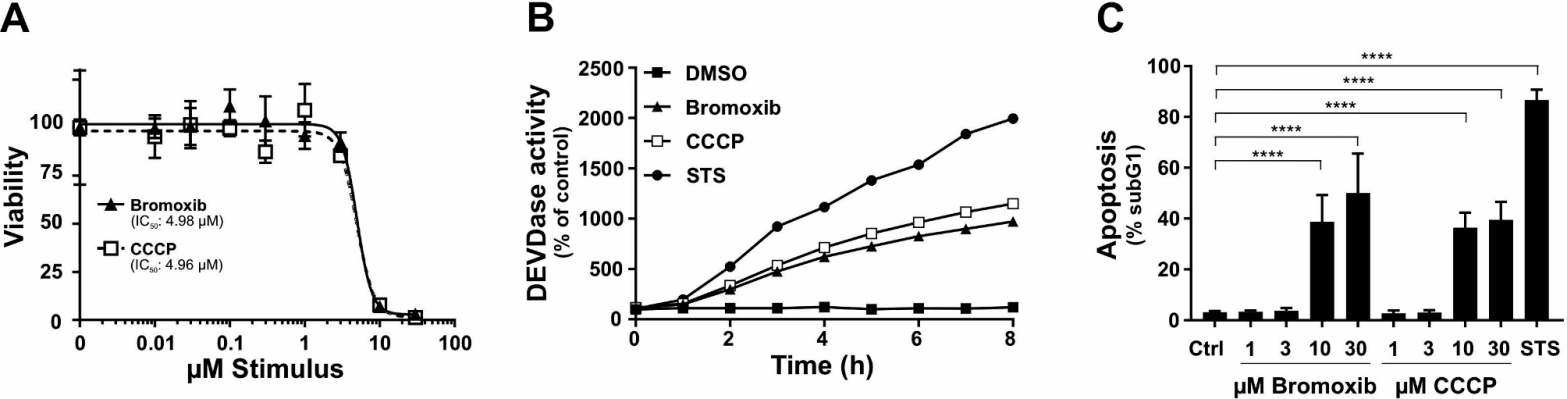
Bromoxib and CCCP display similar cytotoxic features. **(A)** To assess cytotoxicity in Ramos cells, bromoxib, and CCCP were administered and cell viability was determined after 24 h using the AlamarBlue^®^ viability assay. Shown in each graph is the mean ± SD of one representative experiment performed in triplicates. Respective IC_50_ values are shown. **(B)** Ramos cells were subjected to various treatments, including DMSO (0.1% v/v) as a solvent control, bromoxib (10 µM), CCCP (10 µM), or STS (2.5 µM), for a maximum duration of 8 h. To assess caspase 3 activation, DEVDase activity was measured using a spectrofluorometer. The fluorescence increase was recorded for each indicated timepoint over a 2-hour period, and the slope of the linear range of fluorescence increase was used as a parameter to quantify DEVDase activity. The DMSO control values were set to 100 and the normalized relative fold induction, shown as % of control was calculated. Shown in each graph is the mean only of one representative experiment performed in duplicates. **(C)** Ramos cells were exposed to escalating concentrations of bromoxib or CCCP, while DMSO (0.1% v/v) served as solvent control and STS (2.5 µM) as positive control. After 24 h of treatment, apoptosis was assessed by measuring apoptotic nuclei via flow cytometry. Error bars = mean ± SD of three independent experiments performed in triplicates. Statistical analysis: 1-way ANOVA with Dunnett’s multiple comparisons test; (**** = *p* ≤ 0.0001)

Since bromoxib displayed several features of the uncoupler CCCP, we compared bromoxib with other protonophores concerning OCR, cytotoxicity, caspase-activation and apoptosis-induction. Using the Seahorse Mito Stress Test we analyzed the concentration-dependent mitochondrial uncoupling of bromoxib. For the protonophore FCCP it has been shown that it induces a concentration-dependent metabolic demand, leading to rapid oxidation of substrates such as sugars, lipids and amino acids [32]. In the case of bromoxib, we observed a striking similarity when comparing the concentration-dependent, plateau-like elevation of OCR in bromoxib-treated cells with the corresponding increase induced by FCCP-treatment, suggesting a similar cellular response. Thus, bromoxib appears to uncouple mitochondrial respiration in a similar way to FCCP by disrupting the proton gradient, leading to a rapid increase in OCR even at low concentrations (Fig. 5E). The observed subsequent reduction in OCR with a kinetic delay (which cannot be induced even by the addition of FCCP) is fully consistent with the additional inhibition of complexes II, III, and V after prolonged incubation with brmoxib (Fig. 5C, E). Likewise, bromoxib displayed a similar potential as the protonophore CCCP in terms of cytotoxicity, caspase-activation, and apoptosis rate (Fig. 6A, B and **C**). Remarkably, both bromoxib and CCCP displayed similar cytotoxic IC_50_ values in Ramos cells (bromoxib: 4.98 µM; CCCP: 4.96 µM; Fig. 6A). In addition, bromoxib and CCCP initiated the activation of caspases with similar kinetics (within 8 h) and to a similar extent in terms of caspase-activation and apoptosis-induction (Fig. 6B, C). In summary, we demonstrated that bromoxib functions as a mitochondrial uncoupler and induces apoptosis similar to the protonophore CCCP in Ramos cells.

### In thermal proteome profiling (TPP), bromoxib caused the stabilization of tubulin and proteins involved in fatty acid oxidation (FAO)

**Fig. 7:**
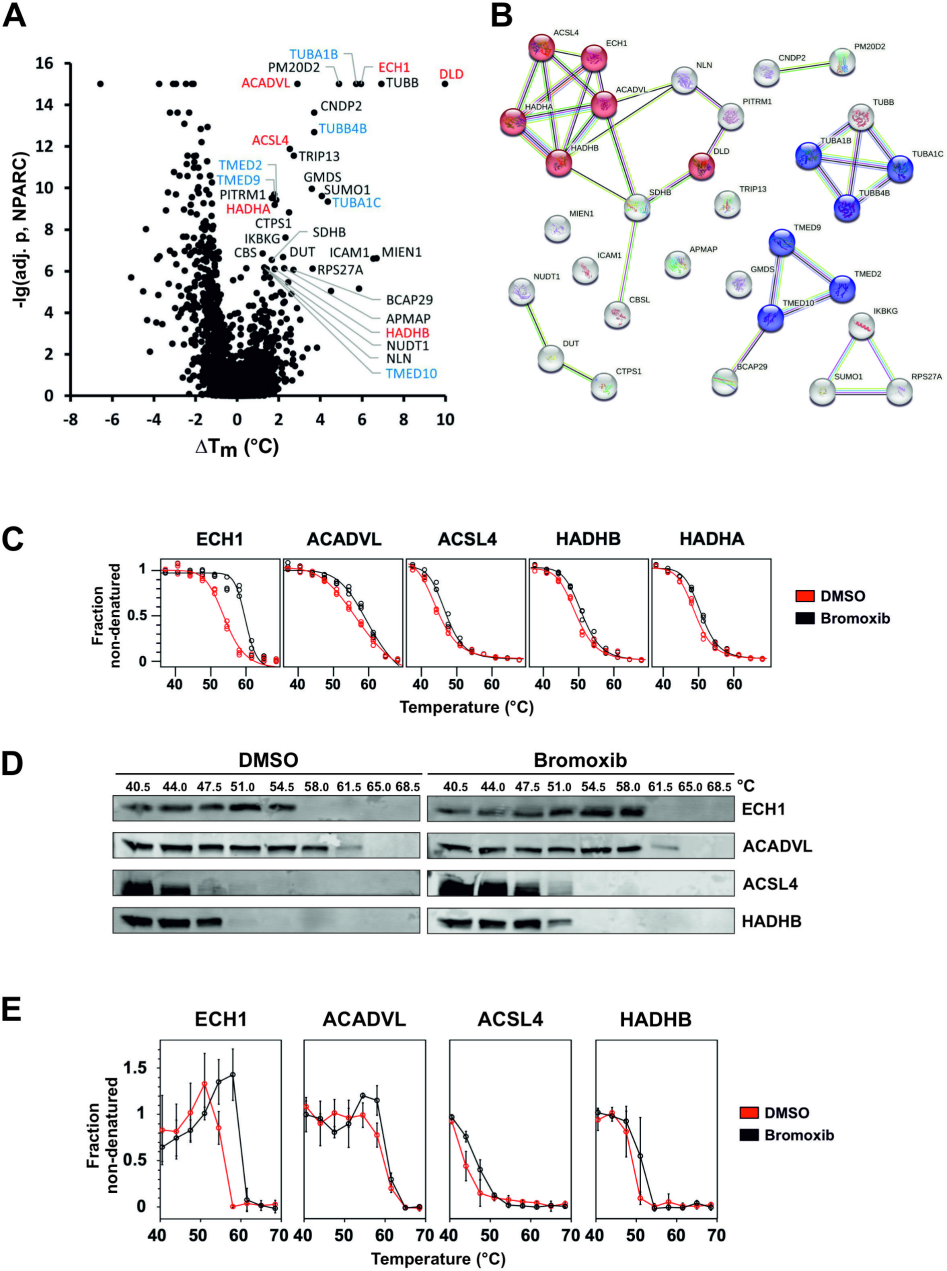
Thermal proteome profiling (TPP) revealed bromoxib-mediated stabilization of multiple proteins. Mass spectrometry based TPP was used to analyze the thermal stabilization and destabilization of proteins upon treatment with bromoxib. For this, Ramos cells were treated with 40 µM bromoxib or a diluent control (DMSO, 0.4% v/v) for a duration of 30 min. **(A)** Volcano-like plot of the statistical significance (expressed as the negative decadic logarithm of the adjusted NPARC p-value, -lg(adj. p, NPARC)) versus the bromoxib mediated melting point shift (difference of the means of the melting points with and without bromoxib treatment, Δ*T*_*m*_) for each protein. Stabilized proteins (cutoffs: -lg(adj. p, NPARC) > 6 and Δ*T*_*m*_ > 0.5°C) are labeled and color coded as in panel B. **(B)** A functional protein association network (based on a STRING database enrichment analysis, https://string-db.org, v11.5) of the selected 31 stabilized proteins from panel A. Proteins related to ‘fatty acid metabolic process’ (GO:0006631) are shown in red and to ‘COPI-mediated transport’ (‘COPI-mediated anterograde transport’: HSA-6807878 and ‘COPI-dependent Golgi-to-ER retrograde traffic’: HSA-6811434) in blue. The colors of the links indicate the type of interactions (light blue: known interactions from curated databases, purple: known experimentally determined interactions, green: predicted interactions by gene neighborhood, red: predicted interactions by gene fusions, predicted interactions by gene co-occurrence, yellow: text mining, black: co-expression, light purple: protein homology). **(C)** The five proteins belonging to the ‘fatty acid metabolism’ with their melting curves: (enoyl-CoA hydratase 1 (ECH1), acyl-CoA dehydrogenase very long chain (ACADVL), acyl-CoA synthetase long chain family member 4 (ACSL4), hydroxyacyl-CoA dehydrogenase trifunctional multienzyme complex subunit β (HADHB) and subunit α (HADHA)). Upon bromoxib treatment (40 µM bromoxib for 30 min), a thermal stabilization of these proteins was identified. **(D)** Exemplary immunoblots (CETSA; see (**E**) for quantitative analysis) against proteins of the ‘fatty acid metabolism’ (ECH1, ACADVL, ACSL4, and HADHB) employing one of the three independent biological replicates used for the TPP. Since HADHB and HADHA belong to the same protein complex whose subunits have different functions, only HADHB is shown. Upon 40 mM bromoxib treatment for 30 min, thermal stabilization of the proteins can be seen in the immunoblots. **(E)** Quantitative analysis of immunoblots (CETSA; see (**D**) for representative examples). The non-denatured fractions of the proteins ECH1, ACADVL, ACSL4, and HADHB from three independent biological replicates (n = 3) were quantified, the normalized signal intensity is shown for DMSO in red and bromoxib in black. Error bars = mean ± SD of three independent biological experiments

In order to identify proteins targeted by bromoxib, we conducted an unbiased proteomic analysis employing thermal proteome profiling (TPP). This method detects drug-protein interactions in living cells by identifying proteins whose thermal stabilities are altered, directly or indirectly, upon drug binding. Through mass spectrometry, the thermal profiles of these affected proteins can be determined, thereby enabling the successful identification of respective drug targets [33, 34]. Using the TPP-approach, 31 stabilized proteins could be identified in Ramos cells which were stabilized upon treatment with 40 µM bromoxib for 30 min (Fig. 7A and Suppl. Table 1). These proteins could be further subdivided into two main hit-clusters: (i) the COPI-mediated transport system, which is composed of tubulin isoforms and (ii) a protein-cluster involved in fatty acid metabolic processes (Fig. 7A, B and Suppl. Table 1).

Tubulin molecules are composed of heterodimers, consisting of α- and β-chains, which arrange themselves in a head-to-tail manner to form protofilaments running along the length of the microtubule structure. Following bromoxib treatment, two α-tubulins, namely TUBA1B and TUBA1C, in addition to the two β-tubulins TUBB and TUBB4B, exhibited enhanced stability in the TPP-approach. Therefore, we investigated whether the cytotoxic mechanism of bromoxib might be due to a disruption of tubulin polymerization. However, using a tubulin polymerization assay we detected no significant influence on the polymerization rate of tubulin and no disturbance of cell cycle progression as would be expected for an antimitotic toxin (Suppl. Figure 2). Consequently, we excluded a disturbed tubulin polymerization as the major mechanism for bromoxib-mediated cytotoxicity.

Next, we focused on the protein cluster associated with fatty acid and lipid metabolism, including ECH1, ACADVL, ACSL4, and HADHA/B (Fig. 7B). Given that bromoxib predominantly targets mitochondria, it was expected that proteins localized within mitochondria would emerge as prominent candidates in the TPP results. As shown in Fig. 7C, these proteins were stabilized in TPP after only 30 min of exposure to bromoxib. To verify this finding, the bromoxib mediated stabilization of the candidate proteins (ECH1, ACADVL, ACSL4, and HADHB) was analyzed by cellular thermal shift assays (CETSA) [35] by subjecting the same temperature-treated samples analyzed by quantitative MS-based TPP to quantitative immunoblotting. CETSA confirmed that bromoxib treatment resulted in thermal stabilization of these proteins (Fig. 7D, E).

### Bromoxib increases fatty acid oxidation

Fatty acid β-oxidation (FAO) represents a central metabolic pathway responsible for the mitochondrial breakdown of long-chain acyl-CoA to acetyl-CoA. The process begins with the activation of long-chain fatty acids via thioesterification (CoA binding), facilitated by acyl-CoA synthetase (ACSL4). Subsequently, these long-chain fatty acids are transported from the cytosol to the inner mitochondrial area through carnitine-acylcarnitine translocase (CACT) and released as acyl-CoA [36]. Importantly, all five proteins that were stabilized by bromoxib treatment in the TPP-approach play a central role in FAO. Their distinct functions within the FAO pathway can be summarized as follows: acyl-CoA dehydrogenase (ACADVL) mediates the oxidation of the acyl-CoA while FAD is reduced to FADH_2_; enoyl-CoA hydratase (ECH1) facilitates hydration; 3-hydroxy acyl-CoA dehydrogenase (HADHA) mediates an additional redox step where NADH is released, and 3-ketoacyl CoA thiolase (HADHB) mediates thiolysis, consequently releasing acetyl-CoA into the TCA cycle and FADH_2_ and NADH into the ETC [36].

**Fig. 8:**
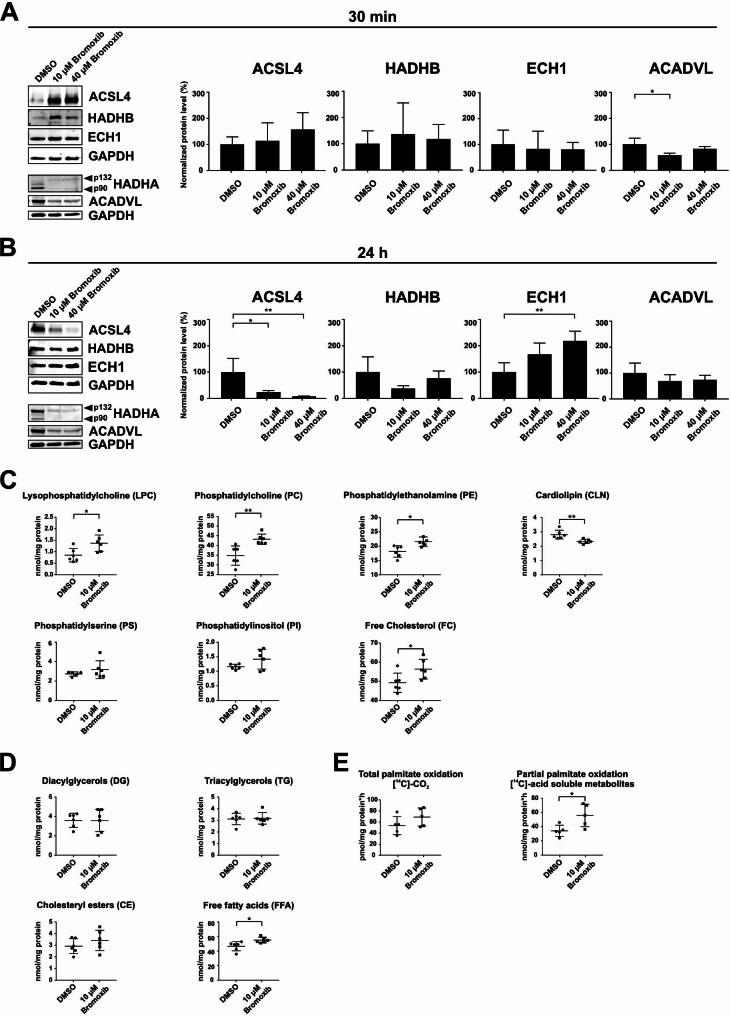
Bromoxib exhibits selectivity towards proteins in the mitochondrial fatty acid β-oxidation pathway, leading to significant changes in the lipid composition of Ramos cells and affecting palmitate oxidation. Ramos cells were treated for **(A)** 30 min and **(B)** 24 h with DMSO (0.1% v/v; negative control), 10 µM or 40 µM bromoxib. The protein levels of the five stabilized proteins of the mitochondrial fatty acid β-oxidation protein cluster: acyl-CoA synthetase long chain family member 4 (ACSL4), hydroxyacyl-CoA dehydrogenase complex subunits A and B (HADHA (p90, p132) and HADHB), enoyl-CoA hydratase (ECH1) and acyl-CoA dehydrogenase very long chain (ACADVL) were determined via immunoblotting. GAPDH served as a loading control. **(A)** and **(B)** on the left side: representative immunoblots of three independent biological replicates are shown. On the right side: the respective quantification of the immunoblots as described in Materials and Methods. Error bars = mean ± SD of three independent experiments. Statistical analysis: 1-way ANOVA with Dunnett’s multiple comparison test; (**** = *p* ≤ 0.0001). In **(C)** and **(D)**, Ramos cells were treated for 30 min with 10 µM bromoxib and DMSO (0.1% v/v; solvent control) and lipidomics was performed for the lysates (six biological replicates) on: **(C)** lysophosphatidylcholine (LPC), phosphatidylcholine (PC), phosphatidylethanolamine (PE), cardiolipin (CLN), phosphatidylserine (PS), phosphatidylinositol (PI) and free cholesterol (FC). The lysates were also analyzed for the different fatty acids **(D)** such as diacylglycerols (DGs), triacylglycerols (TGs), cholesteryl esters (CE) and free fatty acids (FFA). Error bars = mean ± SD of six independent experiments. In **(E)**, the total and partial palmitate oxidation were analyzed in Ramos cells treated with DMSO (0.1% v/v), 10 µM bromoxib (five biological replicates) for 30 min. [^14^C]-CO_2_ as well as [^14^C]-acid soluble metabolites were detected after metabolization as measures for the total and partial palmitate oxidation. Error bars = mean ± SD of five independent experiments. Statistical analysis: unpaired two-tailed t-test; (**** = *p* ≤ 0.0001)

Therefore, we investigated the effect of bromoxib on fatty acid β-oxidation. First, we analyzed to what extent the protein levels of ECH1, ACADVL, ACSL4, and HADHA/B were affected upon treatment with bromoxib for 30 min–24 h. Quantification of immunoblots revealed no significant change in protein levels of ECH1, ACADVL, ACSL4, and HADHB after 30 min treatment with 40 µM bromoxib (Fig. 8A). However, protein level analysis upon 24 h treatment with bromoxib showed a noticeable reduction of ACSL4 and HADHB, an insignificant reduction of ACADVL levels, and a significant increase in ECH1 levels (Fig. 8B). This difference between short-term and long-term treatment may be attributed to different rapid and delayed response mechanisms (such as mitophagy at 24 h).

Next, we investigated the effect of bromoxib on fatty acid metabolism by analyzing the total lipid content (Fig. 8C and **D**) and of the total and partial palmitate oxidation in Ramos cells via the detection of [^14^C]-CO_2_ (total palmitate oxidation) and [^14^C]-acid-soluble metabolites (partial palmitate oxidation) (Fig. 8E). Fatty acids (FAs) serve as building components of various types of lipids. They contain a carboxylic acid moiety coupled with a hydrocarbon chain characterized by varying carbon lengths and degrees of saturation [37]. By channeling FAs into different metabolic pathways, it is possible to produce more complex lipid species such as diacylglycerols (DGs), triacylglycerols (TGs), and glycerophospholipids including phosphatidylethanolamine (PE), phosphatidylinositol (PI), phosphatidylcholine (PC), lysophosphatidylcholine (LPC), and phosphatidylserine (PS), among others [38]. Additionally, FAs contribute to the production of triacylglycerols (TGs) and cholesteryl esters (CEs), which serve as reservoirs of energy in form of lipid droplets. When needed, these lipid droplets are used as a fuel source for cellular bioenergetics via fatty acid oxidation [39, 40]. Free FAs undergo esterification in the glycerol phosphate pathway, giving them amphiphilic properties similar to phospholipids and sphingolipids. In particular, cholesterol, phospholipids, and sphingolipids constitute the basic building blocks of biological membranes [39, 41].

Following treatment with 10 µM bromoxib for 30 min, we observed a significant elevation in the levels of lysophosphatidylcholine, phosphatidylcholine, phosphatidylethanolamine, and free cholesterol. Conversely, cardiolipin levels decreased significantly (Fig. 8C). Diacylglycerols, cholesteryl esters and triacylglycerols were unaffected, whereas free fatty acids were increased upon bromoxib treatment. (Fig. 8D). In terms of total and partial palmitate oxidation (as surrogate markers for FAO activity), a significant increase in [^14^C]-acid-soluble metabolites was observed following treatment with bromoxib, indicating an increase in partial palmitate oxidation (Fig. 8E). Thus, it appears that the cell attempts to compensate for the loss of ATP production (due to the breakdown of OXPOS) by rapidly upregulating FAO upon bromoxib-treatment.

## Discussion

We previously identified the polybrominated diphenyl ether bromoxib in a screen from a library of 300 different natural compounds, where it displayed a strong cytotoxic potential in several leukemia and lymphoma cell lines (Jurkat, THP1, HL60 and Ramos). In addition, bromoxib showed a 3.2-fold lower IC_50_ value in primary leukemia cells from patients with acute myeloid leukemia (AML) compared to the PBMNC from healthy donors [6]. This has prompted us to investigate the molecular mechanism of bromoxib-induced cell death in more detail. Here, we show that bromoxib has a pronounced cytotoxic potential primarily in leukemia and lymphoma cell lines (such as HL60, HPBALL, Jurkat, K562, KOPTK1, MOLT4, SUPB15 and Ramos), but also in solid tumor cell lines such as the glioblastoma cell lines SJ-GBM2 and TP365MG, whereas all other solid tumor cell lines were less sensitive (Fig. 2A, B).

Furthermore, we showed that bromoxib activated the intrinsic mitochondrial apoptosis pathway since it induced the mitochondrial translocation of Bax and mitochondrial release of Smac within 1–2 h (Fig. 3). In addition, bromoxib-induced apoptosis was completely blocked in Jurkat cells deficient for caspase 9 or Jurkat cells overexpressing antiapoptotic Bcl-2 (Fig. 2D, E). Targeting of the mitochondria was further corroborated as bromoxib induced a rapid breakdown (within 5 min) of the ΔΨm in similar kinetics as the protonophore CCCP (Fig. 4A). In addition, bromoxib and CCCP induced the reversible cleavage of L-OPA1 into S-OPA1 forms which coincided with the rapid and pronounced fragmentation of the mitochondrial network within 30 min (Fig. 4B, D).

The mitochondrial fragmentation and the cleavage of L-OPA1 appeared to be mediated by the metallopeptidase OMA1 rather than YME1L1 (Fig. 4C). Both proteases function in different physiological settings. OMA1 is activated by diverse stress conditions, such as loss of ΔΨm, heat stress, or oxidative stress [42–45]. It converts all L-OPA1 isoforms into the shorter version, leading to the fission of the inner mitochondrial membrane (IMM) and subsequent fragmentation of the mitochondrial network. YME1L1, on the other hand, is the principal protease governing L-OPA1 processing under non-stress conditions and is essential for maintaining an equilibrium between mitochondrial fusion and fission [21, 22, 42, 45, 46]. Using murine embryonic fibroblasts deficient for OMA1, YME1L1, or double knockouts, we showed that both bromoxib- and CCCP-induced processing L-OPA1 was dependent on OMA1 (Fig. 4C), which was most likely activated by the breakdown of ΔΨm.

However, bromoxib displayed an additional feature that might contribute to mitochondrial fission, as bromoxib induced the rapid release of Ca^2+^ both from the ER, but apparently also from other stores such as mitochondria, since pretreatment with thapsigargin (which causes a net efflux of Ca^2+^ from the ER) did not completely abolish subsequent bromoxib-induced Ca^2+^-release (Fig. 4F). An increase in cytosolic Ca^2+^ activates the Ca^2+^-dependent phosphatase calcineurin, which in turn dephosphorylates DRP1, leading to its association with mitochondria and subsequent fission [24, 25]. In addition, the proapoptotic proteins Bax and Bak also contribute to mitochondrial fragmentation by stabilizing the mitochondrial association of DRP1 [47].

Intriguingly, bromoxib shared several features with the protonophore CCCP, such as the rapid breakdown of ΔΨm, OMA1-dependent processing of OPA1 and subsequent fission. Both drugs induced caspase-activation in similar kinetics and concentration dependencies in terms of cytotoxicity and apoptosis (Fig. 6A-C). Thus, bromoxib functions as an uncoupler similar to the protonophore CCCP, which inhibits mitochondrial OXPHOS by dissipating the electrochemical proton gradient. In case of CCCP, this is achieved through the transport of protons across the inner mitochondrial membrane (IMM) and thereby disrupting the proton gradient. As a result, the membrane is depolarized, ATP production is reduced, and the cellular metabolism is disrupted, ultimately leading to cell death [48, 49].

However, bromoxib displayed additional features to the protonophore CCCP. In contrast to CCCP, which inhibited OXPHOS-mediated ATP formation, bromoxib also reduced ATP levels generated by glycolysis (Fig. 5A). In addition, protonophores such as CCCP or FCCP inhibit OXPHOS-mediated ATP production by uncoupling the formation of the electrochemical proton gradient from ATP synthesis, but they do not target individual complexes of the ETC. Bromoxib however, inhibited ETC complexes II, III and V (ATP-synthase; Fig. 5B) in addition to its feature as an uncoupler.

In order to identify potential targets of bromoxib we performed a mass spectrometric thermal protein profiling (TPP) approach. Thus, we could identify a cluster of proteins involved in fatty acid oxidation (FAO). This cluster involved the enzymes ECH1, ACADVL, ACSL4, and HADHA/B (Fig. 7, Suppl. Table 1). This finding was corroborated through various methods, including immunoblotting, lipidomics, and the measurement of fatty acid oxidation, confirming the FAO-protein cluster as a metabolic target influenced by bromoxib (Fig. 8). Unexpectedly, bromoxib did not inhibit FAO, but rather increased it, as evidenced by an increase in palmitate oxidation (Fig. 8E). However, since multiple proteins in this metabolic pathway were affected, a direct interaction between bromoxib and any single protein is unlikely. In addition, a direct interaction would inhibit rather than increase the activity of the respective enzymes. Therefore, the stabilization of this protein cluster could rather be a cellular response to the mitochondrial damage caused by bromoxib treatment. Thus, in a futile attempt to compensate for the bromoxib-mediated inhibition of ATP generation (due to impairment of the ETC, OXPHOS and glycolysis), the cell responds by increasing the metabolic activity of the FAO pathway.

Multiple studies indicate that mitochondrial metabolism and OXPHOS contribute, at least partially, to chemoresistance in cancer. Accordingly, targeting of the mitochondrial metabolism has emerged as a promising strategy for overcoming therapy resistance in cancer [2, 50]. In this context, different mitochondrial uncouplers such as CCCP, FCCP and 2,3-dinitrophenol have been tested in vitro for their anticancer potential in different leukemia, lymphoma, as well as solid tumor models [51–65]. Other mitochondrial uncouplers such as niclosamide were also tested in vivo in human glioblastoma (pGBMs) xenografts [66], oesophageal cancer xenografts (ESO26 and ESO26-PAC) [67], adrenocortical carcinoma xenografts (NCI-H295R) [68, 69], non-small lung cancer xenografts (CL1-5) [70], breast cancer C57BL/6 model (E00771) [71], B16F0 mouse melanoma syngeneic model [72], MC38 allografts in NSG mice [73], and WSU-HN6 xenografts [74] (further uncouplers are described in [75]). For more than five decades, the FDA-approved compound niclosamide has been used to treat tapeworm infections and functions as a mitochondrial uncoupler [76]. Studies have now shown that niclosamide exhibits antineoplastic activity against various cancers, including leukemia, kidney, ovarian, colon, melanoma, non-small cell lung, prostate, and breast cancer [77]. Niclosamide is currently tested in clinical trials for pediatric patients with relapsed and refractory AML [recruiting; NCT05188170] and patients with hormone-resistant prostate cancer (active; not recruiting; NCT02807805).

Another interesting feature of bromoxib is its high toxicity in leukemia and lymphoma cells (such as HL60, HPBALL, Jurkat, K562, KOPTK1, MOLT4, SUPB15 and Ramos; Fig. 2A), whereas (with the exception of the glioblastoma cell lines SJ-GBM2 and TP365MG) all other solid tumor cell lines tested (HeLa, MCF7, RT112, LN209, LN308, and YKG1) were much less susceptible (Fig. 2B).

One explanation for this phenomenon may be, that leukemia are characterized by an increased mitochondrial metabolism, which renders them resistant to conventional genotoxic therapies as well as targeted therapies [78, 79]. Accordingly, among the various cancer entities, leukemia and lymphoma appear to be particularly susceptible to targeting of the mitochondrial metabolism [2, 80–82].

In conclusion, we show that bromoxib displays uncoupling properties but also inhibits ATP production from glycolysis and mitochondrial OXPHOS by directly targeting ETC complexes II, III and V (ATP-synthase). Since bromoxib exhibited preferential cytotoxicity against leukemia and lymphoma cells and displayed a therapeutic window in healthy peripheral mononuclear blood cells compared to primary patient AML samples [6], it represents a promising agent for the treatment of blood cancers.

## Materials and methods

### Extraction and isolation of bromoxib

Bromoxib (4,5,6-tribromo-2-(2’,4’-dibromophenoxy) phenol; formerly termed P01F08 [6, 7]) was obtained from the biobank (natural compound library) of the Institute for Pharmaceutical Biology and Biotechnology of the Heinrich Heine University Düsseldorf. Bromoxib was freshly prepared and dissolved in DMSO at 10 mM stock solution. Until use for the assays, the compound was kept at -20 °C in a temperature-controlled refrigerator.

### Reagents

Staurosporine (#9300) was obtained from LC Laboratories (Woburn, MA, USA), *N*-(2-quinolyl)-valyl-aspartyl-(2,6-difluorophenoxy) methyl ketone (QVD) (#S7311) from Selleckchem (Houston, TX, USA) and etoposide (#1043 − 100) from BioVision. SUPERKILLER TRAIL^®^ Protein (soluble) was obtained from human Enzo Life Sciences (#ALX-201-115-C010). Ionomycin (#10634), thapsigargin (#T9033), cyanide *m*-chlorophenyl hydrazine (CCCP) (#2759), rotenone (#45656), thenoyltrifluoroacetone (TTFA) (#88300), antimycin A (#8674), sodium azide (NaN_3_) (#S2002), paclitaxel (#T7402), nocodazole (#M1404), vinblastine (VBL) (#V1377), and CD95-ligand (CD95L/Apo-1 L/FasL; #SRP3036) were obtained from Sigma (Munich, Germany), oligomycin from Toronto Research Chemicals (Toronto, Canada) (#O532970), potassium cyanide (KCN) was supplied in the MitoCheck^®^ kit (Cayman Chemicals; Ann Arbor, Michigan, USA), and the ETC complex II-inhibitor 3-bromopyruvate (3-BP) was obtained from Merck-Millipore (Darmstadt, Germany) (#376817). All other substances for which a manufacturer is not explicitly specified were obtained from Carl Roth (Karlsruhe, Germany).

### Cell lines and cell culture

Ramos cells (human B cell Burkitt lymphoma) were kindly provided by Michael Engelke (Institute of Cellular and Molecular Immunology, University Hospital Göttingen, Germany). Stable transfectants of Jurkat cells (human T cell acute lymphoblastic leukemia) with Bcl-2 overexpression and corresponding empty vector control cells were kindly provided by Claus Belka [17, 83] (Department of Radiation Oncology, University Hospital, LMU Munich, Germany). Jurkat cells deficient for caspase 8 and the parental cell line A3 [13, 17] were kindly provided by John Blenis (Sandra and Edward Meyer Cancer Center, New York, NY, USA). Jurkat cells deficient for caspase 9 were kindly provided by Klaus Schulze-Osthoff (Interfaculty Institute for Biochemistry, University of Tübingen, Germany) [84] and retrovirally transduced with either empty pMSCVpuro (Clontech, Heidelberg, Germany) or pMSCVpuro containing cDNAs coding for untagged human wild-type caspase 9 as previously described [15, 17]. MCF7 (human breast carcinoma; #ACC-115), RT112 (human urinary bladder carcinoma; #ACC-418), HL60 (human acute myeloid leukemia; #ACC-3), HPBALL (human T cell acute lymphoblastic leukemia; #ACC-483), MOLT4 (human T cell acute lymphoblastic leukemia; #ACC-362), K562 (human chronic myeloid leukemia; #ACC-10) and SUPB15 (human B cell acute lymphoblastic leukemia; #ACC-389) were obtained from DSMZ and provided by Sanil Bhatia (Dept. of Pediatric Oncology, Hematology and Clinical Immunology, University Hospital Düsseldorf, Germany). KOPTK1 (human T cell acute lymphoblastic leukemia; CVCL-4965) was kindly provided by Oskar Haas (Children’s Cancer Research Institute, St. Anna Children’s Hospital, Vienna, Austria). HeLa cells stably expressing mito-DsRed were kindly provided by Aviva M. Tolkovsky (Department of Clinical Neurosciences, University of Cambridge, England, UK) and have been described previously [85]. HeLa cells with transient expression of Smac-mCherry and GFP-Bax were generated by lipofection at 70–80% confluence using Lipofectamine 2000 (Life Technologies, Darmstadt, Germany). Therefore, cells were incubated with 0.15 µL Lipofectamine 2000, 50 ng pcDNA3-Smac(1–60)mCherry (Addgene ID 40880; this plasmid was kindly provided by Stephen Tait (Beatson Institute, University of Glasgow, Scotland, UK) and has been described previously [86]) and 50 ng pGFP-Bax (kindly provided by Nathan R. Brady, Department of Molecular Microbiology and Immunology, Johns Hopkins University, Baltimore, MD, USA) per well on µ-Slide 8 wells (IBIDI, Planegg, Germany, #80826) for 16 h as described in [26]. MEF (mouse embryonic fibroblast) cells deficient for OMA1 and/or YME1L1 as well as the corresponding wild-type cells were generated by Ruchika Anand (Institute of Biochemistry and Molecular Biology I, University of Düsseldorf, Germany) and kindly provided by Thomas Langer (Institute for Genetics, University of Cologne, Germany) [21]. The glioblastoma cell line LN229 (#CRL-2611) was obtained from ATCC. The glioblastoma cell lines LN308 and TP365MG were kindly provided by Guido Reifenberger (Inst. of Neuropathology, University Hospital Düsseldorf, Germany). The glioblastoma cell line SJ-GBM2 was obtained from Children’s Oncology Group (Dept. of Hematology, Oncology and Clinical Immunology, University Hospital Düsseldorf, Germany) and YKG1 from Tebu-Bio (ref JCRB0746). All cells were cultivated at 5% CO_2_ at 37 °C and stable humidity. The suspension cell lines (HL60, HPBALL, Jurkat, KOPTK1, K562, MOLT4, Ramos, SUPB15) and MCF7 were maintained in RPMI media with 10% FCS, 100 U/mL penicillin and 100 µg/mL streptomycin. MEF cells, RT112, HeLa, YKG1, TP365MG, LN229 and LN308 were maintained in high-glucose Dulbecco’s Modified Eagle’s medium (DMEM) supplemented with 10% FCS, 100 U/mL penicillin and 100 µg/mL streptomycin. SJ-GBM2 was cultivated in IMEM medium supplemented with 10% FCS, 100 U/mL penicillin and 100 µg/mL streptomycin.

### Cytotoxicity measurements

To determine cytotoxicity, the AlamarBlue^®^ assay was performed as described in [87]. Briefly, suspension cells were seeded at a density of 1 × 10^6^ cells/mL and adherent cells were seeded at a density of 0.2 × 10^6^/mL. Suspension cells were immediately treated; adherent cells were treated the next day, with increasing concentration of the compound and after the specified incubation time, resazurin (Sigma, #R7017) was added to a final concentration of 40 µM. After 120 min of incubation, the fluorescence of resorufin (excitation (Ex): 535 nm, emission (Em); 590 nm) was measured with a microplate spectrophotometer (Synergy Mix plate reader). The viability of cells treated with DMSO (0.1% v/v) was set to 100% and the dose-response curves were then fitted with PRISM v7.01 (GraphPad Software, La Jolla, CA, USA).

### Fluorometric caspase 3 activity assay

Cells were seeded at a density of 1 × 10^6^ cells/mL in a 96-well plate, treated with the compound for the depicted time (kinetics 0–8 h) and harvested by centrifugation at 600 g. The supernatant was removed and the cells were lysed with 50 µL of ice-cold lysis buffer (20 mM HEPES, 84 mM KCl, 10 mM MgCl_2_, 200 µM EDTA, 200 µM EGTA, 0.5% NP40, 1 µg/mL leupeptin, 1 µg/mL pepstatin, 5 µg/mL aprotinin) on ice for 10 min. The lysates were transferred to a flat-bottom microplate and mixed with 150 µL ice-cold reaction buffer (50 mM HEPES, 100 mM NaCl), 10% sucrose, 0.1% CHAPS, 2 mM CaCl_2_, 13.35 mM DTT) containing 70 µM of the profluorescent caspase substrate Ac-DEVD-AMC (Biomol GmbH, Hamburg, Germany, #ABD-13420). The kinetics of AMC release were monitored by measuring AMC fluorescence intensity (Ex: 360 nm; Em: 450 nm) at 37 °C every 2 min over a total of 120 min, using a Synergy Mix microplate reader. The slope of the linear range of the fluorescence curves (Δrfu/min) was considered as corresponding to caspase 3 activity.

### FACS-based analysis of apoptotic cell death and cell cycle

Fragmented DNA leaking from apoptotic nuclei and the cell cycle was measured by the method of Nicoletti et al. [14]. Briefly, nuclei were prepared by lysing cells in a hypotonic lysis buffer (1% sodium citrate, 0.1% Triton X-100, 50 µg/mL propidium iodide) and subsequently analyzed by flowcytometry. For analysis of hypodiploid apoptotic nuclei (which are located to the left of the 2 N-peak), nuclei were measured in logarithmic mode. For cell cycle analysis, nuclei were measured in linear mode to distinguish G_1_, S, G_2_ phases and hypodiploid nuclei. All flowcytometry analyses were performed on an LSR-Fortessa™ (Becton, Dickinson, Heidelberg, Germany). Data analysis was performed using FlowJo_V10 (BD Biosciences). For statistical analysis of hypodiploid nuclei, 1-way ANOVA with Dunnett’s multiple comparison’s test was performed comparing treatments to DMSO (0.1% v/v). **** *p* < 0.0001.

### Immunoblotting

Cells were seeded at a density of 1 × 10^6^ cells/mL, treated as specified and harvested by centrifugation (3000 rpm, 5 min) followed by freezing in liquid nitrogen. The cell pellets were thawed on ice followed by three quick-freeze-cycles in liquid nitrogen. Subsequently, the pellets were lysed with lysis buffer (20 mM Tris-HCl, 150 mM NaCl, 1% v/v Triton X-100, 0.5 mM EDTA, 10 mM NaF, 2.5 mM Na_4_P_2_O_7_, 0.5% sodium deoxycholate and protease inhibitors (Sigma, #P2714)) on ice for 30 min, accompanied by vortexing. The lysates were purified from cell debris by centrifugation (13300 rpm, 15 min). Protein concentration was determined with Bradford assay. Samples were diluted in lysis buffer and sample buffer, followed by SDS-PAGE and Western blotting according to standard workflows. Finally, target-specific antibodies (anti-PARP 1:2000 (Enzo, #BML-SA250); anti-GAPDH 1:5000 (Abcam, #ab8245), anti-α-tubulin 1:2000 (TUBA4A; Sigma, #T5168), anti-OPA1 1:1000 [88], anti-OXPHOS WB antibody cocktail 1:500 (Abcam, #ab110411, consisting of anti-NDUFB8 (NADH ubiquinone oxidoreductase subunit B8), anti-SDHB (succinate dehydrogenase subunit B), anti-UQCRC2 (ubiquinol-cytochrome c reductase core protein 2), anti-cox-2 (cytochrome c oxidase-2) and anti-ATP5A (ATP-synthase F1α)) and anti-vinculin 1:2000 (Sigma, #V9131); anti-caspase 8 (Cell Signaling Technology, #9746), anti-caspase 9 [15], anti-HADHA 1:1000 (Santa Cruz, # sc-374497), anti-HADHB 1:1000 (Santa Cruz, # sc-271495), anti-ACSL4 1:1000 (Santa Cruz, #sc-365230), anti-ECH11:1000 (Santa Cruz, #sc-515270) and anti-ACADVL 1:1000 (Santa Cruz, #sc-376239) were used for immunoblotting. Fluorescence-coupled secondary antibodies (LI-COR Biosciences) were used for the detection of target proteins on PVDF membrane using LI-COR Odyssey^®^ imaging system. Validation of bromoxib mediated thermal stabilization of ECH1, ACADVL, ACSL4, and HADHB by quantitative immunoblotting (CETSA) [35] was essentially performed as described [89]. A detailed description is provided in the Supplemental Information.

### Densiometric analyses

Immunoblots were quantified using the LI-COR software Image Studio Lite Ver 5.2. The densiometric signals from the protein bands of interest were determined. The shown values represent the calculated ratio of the two long forms of OPA1 (L1 plus L2) to total OPA1 (L1 plus L2 plus S3-5).

### Microscopy and quantification of mitochondrial fragmentation (fission)

Imaging of HeLa cells stably expressing mito-DsRed was performed using a Spinning Disc Confocal microscope (Perkin-Elmer, Waltham, MA, USA) equipped with a 60x/1.4 NA oil-immersion objective using a 561 nm laser line for excitation. Approximately 50 planes of 0.3 μm distance were recorded. The cells were seeded on glass bottom 3 cm dishes (MatTek, Ashland, MA, USA) and maintained in full growth medium supplemented with 10 mM HEPES during imaging. Images were taken for a duration of 30 min and 120 min in a chamber heated to 37 °C. Image analysis was performed using Fiji (V 1.53f51). 3D stacks of 50 planes in thickness were compressed with maximum intensity projection and brightness was adjusted for optimal visibility of mitochondria. Imaging files were blinded by renaming files with random numbers and all imaged cells were categorized into three categories (tubular, intermediate, and fragmented). Statistical significance was analyzed by 2-way ANOVA followed by Dunnett’s multiple comparison test. **** *p* ≤ 0.0001.

### Confocal microscopy – live imaging of Smac and Bax intracellular localization

Confocal microscopy live imaging of HeLa wild type cells transiently expressing GFP-Bax and Smac-mCherry was performed using an SP8 Leica microscope (Inverse DMI 6000 AFC, Leica) with a C-Apochromat × 63 N.A. 1.2 water DIC objective. Cells were seeded onto µ-Slide 8 wells (IBIDI, 80826) and transfected with GFP-Bax and Smac-mCherry as described in [26]. Cells were maintained in full growth medium at 37 °C and 5% CO_2_ during imaging and recorded every 5–15 min during 120 min and processed with Fiji as described in [17]. For each individual time frame, we performed a maximum intensity projection, smoothing of the images and measured the standard deviation (SD) of the fluorescence intensity of GFP-Bax and Smac-mCherry channels, to analyze their subcellular distribution upon DMSO (10 µM) or bromoxib treatment (10 µM). To avoid downstream caspase-mediated effects on cell morphology (such as membrane blebbing), 10 µM of the caspase-inhibitor QVD was added prior to incubation with DMSO or bromoxib.

### Live measurement of mitochondrial membrane potential

To evaluate changes in mitochondrial membrane potential (ΔΨm), Ramos cells (1 × 10^6^ c/mL) underwent treatment with the cell-permeable, positively charged dye tetramethylrhodamine ethyl ester (TMRE). This dye tends to accumulate in active mitochondria characterized by a negative net charge, while depolarized mitochondria fail to retain the dye. To perform this, cells were suspended in fresh medium with 10 mM HEPES and supplemented with 100 nM TMRE (AAT Bioquest, Sunnyvale, CA, USA; #22220). After a 15-min incubation at 37 °C, the cells were washed twice with RPMI (supplemented with HEPES) and then given an additional 15 min for recovery to ensure dye accumulation in active mitochondria. Subsequently, TMRE fluorescence was measured via flow cytometry (Ex: 488, Em: 575) using LSR-FortessaTM (Becton, Dickinson, Heidelberg, Germany). The initial fluorescence level was recorded for each sample over 2 min before introducing the test substance. The fluorescence level prior to substance introduction was set to 100%. As a positive control for inducing the complete breakdown of ΔΨm, treatment with the protonophore CCCP was employed.

### Live measurement of total and intracellular calcium mobilization by Fluo-4-AM

For the detection of changes in Ca^2+^ homeostasis, Ramos cells (1 × 10^6^ c/mL) were stained by incubation in a growth medium containing 1 µM Fluo-4-AM (Life Technologies; #F14201), 0.005% w/v Pluronic F-127 (Sigma, #540025), 10 mM HEPES and 5% v/v FCS at 30 °C for 25 min. An equal amount of full-growth medium was added and the samples were incubated at 37 °C for 10 min. The cells were then pelleted at 3000 rpm for 3 min and resuspended in Krebs-Ringer buffer (10 mM HEPES pH 7.0, 140 mM NaCl, 4 mM KCl, 1 mM MgCl_2_, 10 mM glucose) supplemented with 1 mM CaCl_2_. The cells were kept in the dark until measurement at RT. Immediately prior to measurement, cells were washed and resuspended in Krebs-Ringer buffer alone for total Ca^2+^ mobilization. For the measurement of intracellular Ca^2+^ mobilization, cells were washed with Krebs-Ringer buffer supplemented with 0.5 mM EGTA. Fluo-4-AM fluorescence was measured live using flow-cytometer LSR-Fortessa™ (Becton, Dickinson, Heidelberg, Germany) recording fluorescence in the FITC channel (Ex: 488, Em: 530 ± 30 nm). For each sample, after at least 30 s of baseline measurement, the stimulus was added and measurement was continued for the indicated time.

### Measurement of cellular ROS level

To measure alterations in cellular reactive oxygen species (ROS) levels, Ramos cells were cultured in phenol red-free RPMI medium throughout the assay. The cells were loaded with 20 µM 2’,7’-dichlorodihydrofluorescein diacetate (H_2_DCF-DA, Sigma, #D6883) and incubated for 1 h in the dark at 37 °C. Subsequently, the cells were centrifuged (600 g x 5 min) and washed with media, followed by the treatment of cells with desired compounds for 30 min at 37 °C. The cells were centrifuged (600 g; 5 min), washed with PBS, and transferred to FACS tubes. The fluorescence of DCF, which correlates with the cellular ROS level, was measured by flowcytometry at Ex: 488 nm, Em: 530 nm (FITC channel) at LSR-Fortessa™ (Becton, Dickinson, Heidelberg, Germany).

### Measurement of cellular ATP-levels

For the detection of changes in cellular ATP-levels, the mitochondrial ToxGlo™ assay (Promega, Mannheim, Germany; #G8000) was performed according to the manufacturer’s instructions. To enhance the detection sensitivity of mitotoxins, cells were cultured in a medium containing either glucose or galactose as the sole available sugar source. The utilization of galactose through glycolysis does not result in a net gain of ATP, thereby forcing the cells to rely entirely on OXPHOS for ATP production. The statistical analysis was performed using 2-way ANOVA and Sidak’s multiple comparisons test. **** *p* ≤ 0.0001. The ATP-assay was measured with a microplate spectrophotometer (Synergy Mix plate reader).

#### Measurement of ETC complex (I-V) activity

For the detection of the activity of complex I-V of the ETC, the measurements were performed using the MitoCheck^®^ complex I-V activity assay kits (Cayman Chemical, Ann Arbor, MI, USA; #700930/700940/700950/700990/701000) according to manufacturer’s instructions. The complex activity was measured with a microplate spectrophotometer (Synergy Mix plate reader). For each complex an appropriate positive control for the inhibition was used and the activity of the negative control (DMSO 0.1% v/v) was set to 100%. The statistical analysis was performed using an unpaired t-test comparing treatment to DMSO (0.1% v/v). **** *p* < 0. 0001.

### Seahorse analysis

The Seahorse XFe96 Extracellular Flux Analyzer (Agilent Technologies, Santa Clara, CA, USA) was employed alongside the Mito Stress Test Kit (Agilent Technologies, Santa Clara, CA, USA) following the manufacturer’s instructions. To induce inhibition of ATP-synthase (complex V) and restrict the electron flow along the ETC, oligomycin was applied, thereby leading to a decline in mitochondrial respiration, as indicated by the OCR. This oligomycin induced reduction in OCR is a surrogate marker for ATP production. Subsequently, a decoupling agent, FCCP (carbonyl cyanide-4 (trifluoromethoxy) phenylhydrazone; a CCCP analog), was administered. This agent induces the collapse of the proton gradient and disrupts the ΔΨm. As a result, electron flow through the ETC becomes unrestricted and oxygen consumption by complex IV reaches its maximum capacity, resulting in a plateau effect with DMSO between 60 and 80 min. The FCCP-augmented OCR was then used to calculate the spare respiratory capacity (SRC), defined as the difference between maximal and basal respiration. The SRC provides a measure of the cell’s ability to respond to escalated energy demands or stress conditions. The subsequent application of a combination of rotenone (inhibitor of complex I) and antimycin A (inhibitor of complex III) effectively stops mitochondrial respiration and facilitates the calculation of non-mitochondrial respiration driven by mechanisms outside the mitochondria. For each well, injection compounds were utilized at concentrations of 1 µM oligomycin, 1 µM FCCP, and 0.5 µM rotenone/antimycin A. 10,000 HeLa cells were seeded 24 h before Seahorse analysis. Acute injection of bromoxib (10 µM) or DMSO (0.1% v/v) vehicle preceded OCR measurement for three timepoints every 6 min, before progressing with oligomycin injection. In the assay involving various bromoxib concentrations (0.5–10 µM), four timepoints were assessed, each after 3 min. Non-mitochondrial respiration was designated as the lowest recorded OCR after interrupting respiration with rotenone/antimycin A. Mitochondrial respiration parameters were calculated according to Agilent Report generator user guide.

### Lipidomics and measurement of fatty acid oxidation

#### Quantification of lipids

Ramos cells (1.5 × 10^6^ c/mL) were treated for 30 min with 10 µM bromoxib and DMSO (0.1% v/v; solvent control). For lipid quantification, cells were directly suspended in 0.3 mL distilled H_2_O. Lipids were extracted from 0.15 to 0.35 mg of protein, as outlined in [90]. To comprehensively quantify lysophosphatidylcholine (LPC), phosphatidylcholine (PC), phosphatidylserine (PS), phosphatidylinositol (PI), phosphatidylethanolamine (PE), cardiolipin (CLN), free cholesterol (FC), diacylglycerols (DG), triacylglycerols (TG), and cholesteryl esters (CE), thin layer chromatography (TLC) plates were used [91]. These plates were then stained with a solution consisting of 10% CuSO_4_ (w/v) in 8% H_3_PO_4_ (v/v), and the plate image was digitized using the ChemiDocTM PM imaging system (Bio-Rad Laboratories, USA). Quantification was conducted using the Image Lab software (Bio-Rad Laboratories, USA). Free fatty acids were measured using a commercial kit (WAKO Fujifilm; Japan) directly from the cell suspension, following the manufacturer’s protocol. Lipid levels were expressed relative to the cell protein content.

#### Fatty acid oxidation in cells

To assess the rate of fatty acid oxidation in Ramos cells, the quantification of [^14^C]-CO_2_ (indicative of complete oxidation) and [^14^C]-acid-soluble metabolites (ASM) (indicative of incomplete oxidation) released from [1-^14^ C]-palmitate (obtained from Perkin Elmer, USA) oxidation was carried out, following a methodology previously described with slight modifications [92, 93]. In essence, a cell concentration of 1.5 × 10^6^ c/mL was subjected to overnight starvation using assay medium (low glucose MEM supplemented with 0.3 g/L of glutamine, 6 g/L HEPES, 5% penicillin/streptomycin mix (v/v), and 0.5% fatty acid-free BSA (w/v)). Subsequently, the cells were pre-treated for 30 min with either bromoxib (10 or 40 µM) or the vehicle control (DMSO 0.1% (v/v)) within the assay medium. Following this, the cells were incubated for a duration of 4 h with medium supplemented with fatty acid-free BSA complexed with 0.2 mM palmitate containing 0.5 µCi/ml [1-^14^ C]-palmitate. Thereafter, the collected medium was placed in a tube containing Whatman filter paper soaked in 0.1 M NaOH, with 500 µl of 3 M perchloric acid being added to release the CO_2_, which was then captured within the filter paper. The acidified medium was subsequently centrifuged at 21,000 x g for 10 min to eliminate particulate matter. The radioactivity stemming from CO_2_ captured by the filter papers and ASM present in the supernatants of the culture media was quantified using a Tricarb 2810 TR scintillation counter (Perkin Elmer, USA), and the measurements were expressed relative to the cell protein content.

### Tubulin polymerization assay

For the detection of changes in tubulin polymerization, the OD-based tubulin polymerization assay kit (Cytoskeleton, Inc., Denver, CO, USA; #BK006P) was performed according to the manufacturer’s protocol. Based on the observation, that monomeric tubulin polymerization is accompanied by an increase in absorption (340 nm), this underlying reaction can be measured. The velocity of polymerization has been calculated using the formulas provided by the manufacturer. The respective controls for tubulin stabilization (paclitaxel; which increases the polymerization rate) and for tubulin destabilization (nocodazole; which reduces the polymerization rate) were used.

### Thermal proteome profiling (TPP)

Thermal proteome profiling with temperature range (TPP-TR) was essentially performed as described [18, 33, 89, 94] employing 40 µM as final bromoxib concentration and 30 min treatment time on live Ramos cells in three biological replicates (*n* = 3). In brief, aliquots of compound treated and control cells were shortly incubated side-by-side at ten discrete temperatures, equally distributed in a range between 37 °C and 68.5 °C, lysed, and the cell extracts containing the fraction of soluble, non-denatured proteins were obtained by centrifugation. Peptides were obtained by purification and tryptic digestion of the non-denatured proteins using a slightly modified SP3 protocol [95], labeled using TMT 10plex to code for the different temperatures, combined, offline high pH fractionated, and analyzed by LC-MS. In total, eight (high pH fractions per TMT set) times two (TMT sets per replicate) times three (replicates) = 48 samples were analyzed using an Ultimate 3000 Rapid Separation Liquid Chromatography System and a nano-source ESI interface equipped Orbitrap Fusion Lumos Tribrid mass spectrometer operated in synchronous precursor selection (SPS) mode. MS data analysis was performed using MaxQuant version 2.0.3.0, and TPP-TR data were prepared from the TMT reporter ion intensities by a three-step normalization procedure essentially as described [89] using R (R version 4.1.2 on a x86_64-w64-mingw32/x64 (64-bit) platform). Statistical analysis of melting curves was performed essentially as described [18] using the TPP R package version 3.30.0 in R version 4.3.2. (https://bioconductor.org/packages/TPP). Functional protein association network analysis was performed using the STRING version 11.5 online tool (https://string-db.org [96]). A detailed description is provided in the Supplemental Information.

### Replicates and statistical analyses

The experiments were replicated at least three times, and the presented data are representative experiments. Error bars represent the standard deviation. If not stated otherwise, all statistical analyses were conducted using Prism v7.01 (GraphPad Software, La Jolla, CA, USA).

## Electronic supplementary material

Below is the link to the electronic supplementary material.

Supplementary Material 1

## Data Availability

No datasets were generated or analysed during the current study.
