# Supplementary material for: Targeting mitochondrial metabolism by the mitotoxin bromoxib in leukemia and lymphoma cells
Source: Cell Commun Signal. 2024 Nov 12;22:541. doi: 10.1186/s12964-024-01913-2 (PMC11558866; doi:10.1186/s12964-024-01913-2)
Supplement: Supplementary file 1 — Supplementary Material 1 [file 12964_2024_1913_MOESM1_ESM.pdf]

Supplemental Information

Supplemental Figures

Supplemental Figure 1: Bromoxib does not induce generation of reactive oxygen species (ROS)

Supplemental Figure 2: Bromoxib exerts no effect on cell cycle distribution or tubulin polymerization at cytotoxic concentrations

Supplemental Tables

Supplemental Table 1: List of proteins stabilized by bromoxib as identified by TPP which was performed as described in Supplemental Materials and Methods

Supplemental Materials and Methods

Thermal proteome profiling - temperature range (TPP-TR)

Supplemental References

SUPPLEMENTAL FIGURES

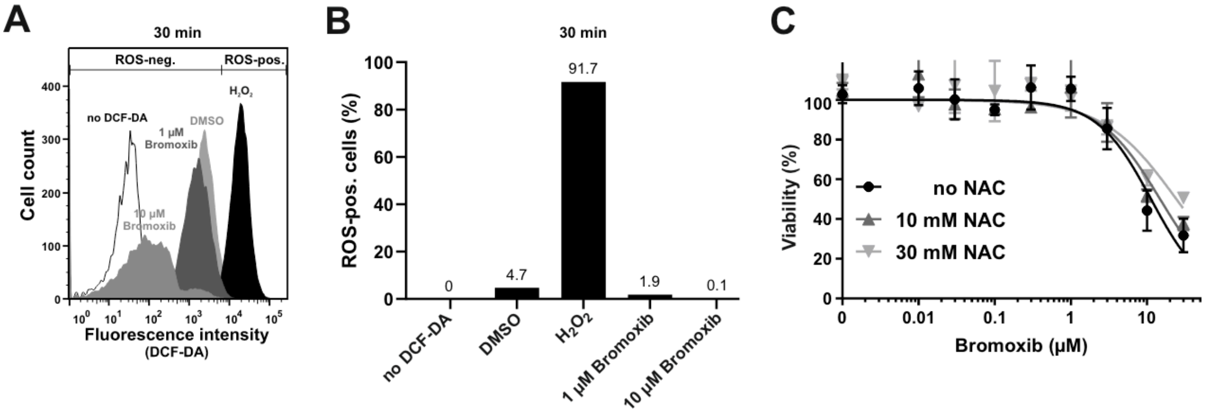

Suppl. Figure 1: Bromoxib does not induce generation of reactive oxygen species (ROS).

(A) The detection of ROS was determined by the DCF-DA assay via flow-cytometric measurement. Ramos cells were treated for 30 min with DMSO (0.1% v/v) as solvent control, 10 mM H_2O_2 as positive control, and 1 or 10 µM bromoxib. **(B)** Quantitative analysis of ROS-positive cells according to the gates set in (A). **(C)** Ramos cells were exposed to escalating concentrations of bromoxib, either alone or in combination with the antioxidant N-acetylcysteine (NAC). Cell viability was assessed using the AlamarBlue® viability assay. Shown in each graph is the mean ± SD of one representative experiment performed in triplicates.

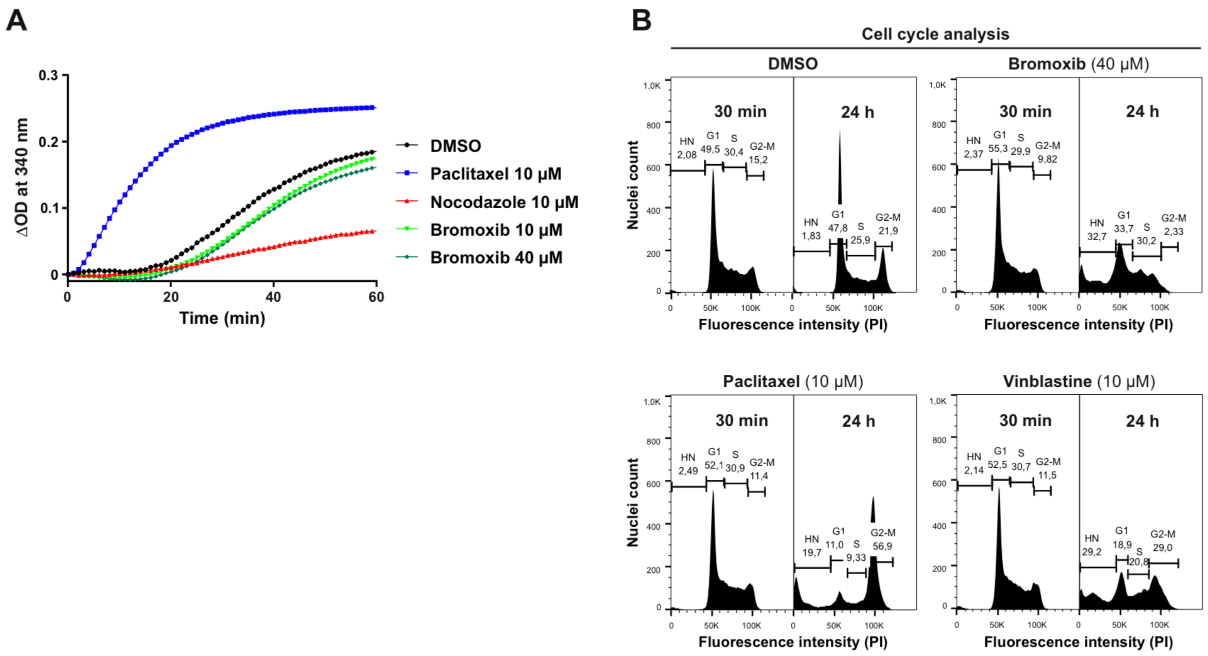

Suppl. Figure 2: Bromoxib exerts no effect on tubulin polymerization or cell cycle distribution at cytotoxic concentrations. (A) Bromoxib has no effect on the tubulin polymerization rate. The tubulin polymerization rate of porcine neuronal tubulin was determined upon treatment with either DMSO (0.1% v/v), paclitaxel (10 µM; as positive control for tubulin stabilization), nocodazole (10 µM; as positive control for tubulin destabilization), or bromoxib (10 or 40 µM). Polymerization was started by incubation at 37 °C and followed by monitoring of the absorption at 340 nm for 60 min. The absorption is proportional to the concentration of the microtubule polymer. The graph shows the mean values of three independent experiments. (**B**) Cell cycle analysis upon bromoxib treatment. Ramos cells were treated for 30 min or 24 h with DMSO (0.1% v/v) as solvent control, bromoxib (40 µM) and paclitaxel (10 µM; as tubulin stabilizing agent) or vinblastine (10 µM; as tubulin destabilizing agent) as positive controls for cell cycle disruption. Cell cycle analysis was performed by propidium iodide staining and flow-cytometric measurement in a linear mode. Cell cycle analysis was performed by propidium iodide staining and flow-cytometric measurement in a linear mode. The different cell cycle phases are shown as representative images of three independent experiments. Bromoxib did not alter the cell cycle distribution.

SUPPLEMENTAL TABLE

Supplemental Table 1: List of proteins stabilized by bromoxib as identified by TPP which was performed as described in the methods section.

Proteins with negative decadic logarithm of the adjusted NPARC p-value (-lg(adj. p, NPARC)) > 6 as measure for statistical significance and melting point difference (ΔT_m) > 0.5 °C were regarded as significantly stabilized (31 proteins). For these proteins, the localization within the cell, as well as the functions and the involved biological processes using Uniprot [1] and Human Protein Atlas [2] are also given.

Gene name(s)	Protein Group IDs from MaxQuant MS analysis software	Protein name(s)	Localization	Protein function and biological process	Δ*Tm* / °C	-lg(adj. p, NPARC)
**ACADVL**	P49748; G3V1M7; K7EQP4; J3QRJ8; J3KS89; J3QKU9; K7EJW8; J3KSR4; K7EMF8	Acyl-CoA dehydrogenase very long chain	Nucleoplasm, Nucleoli, Mitochondria	Very long-chain specific acyl-CoA dehydrogenase is one of the acyl-CoA dehydrogenases that catalyze the first step of mitochondrial fatty acid beta-oxidation, an aerobic process breaking down fatty acids into acetyl-CoA and allowing the production of energy from fats. The first step of fatty acid beta-oxidation consists in the removal of one hydrogen from C-2 and C-3 of the straight-chain fatty acyl-CoA thioester, resulting in the formation of trans-2-enoyl-CoA. **Fatty acid metabolism, Lipid metabolism**	2.9	15*
**ACSL4**	O60488; H0Y9A0; D6RDA8; D6RF95	Acyl-CoA synthetase long chain family member 4	Mitochondria, Golgi apparatus	Catalyzes the conversion of long-chain fatty acids to their active form acyl-CoA for both synthesis of cellular lipids, and degradation via beta-oxidation. **Fatty acid metabolism, Lipid metabolism**	2.5	11.9
**APMAP**	Q9HDC9; H0Y512	Adipocyte plasma membrane-associated protein	Membrane	Exhibits strong arylesterase activity with beta-naphthyl acetate and phenyl acetate. May play a role in adipocyte differentiation.	2.3	6.1
**BCAP29**	A0A087X0G7; A0A087WUW6; A0A087X1B1; Q9Y6K9; A0A087WV30; C9JN51; C9JH59; C9J2V2	Coproporphyrinogen oxidase	Mitochondria	Catalyzes the aerobic oxidative decarboxylation of propionate groups of rings A and B of coproporphyrinogen-III to yield the vinyl groups in protoporphyrinogen-IX and participates to the sixth step in the heme biosynthetic pathway.	2.7	6.0

Gene name(s)	Protein Group IDs from MaxQuant MS analysis software	Protein name(s)	Localization	Protein function and biological process	Δ*Tm* / °C	-lg(adj. p, NPARC)
**CBS**	P0DN79; P35520; H7C2H4; C9JMA6; H7C1W6; H7C2W0	Transmembrane emp24 domain-containing protein 2	ER, Golgi apparatus	Involved in vesicular protein trafficking. Mainly functions in the early secretory pathway but also in post-Golgi membranes.	1.2	6.8
**CNDP2**	Q96KP4; J3QKT2; A0A087WYZ1; J3KSV5; J3QRD0; J3QL02; J3QRA8; J3QKQ0; J3QLU1; J3QR27; J3QQN6; A0A087WVS2; J3QRH4; J3KRD5; J3KSS4; J3KRJ8; J3QRP4	Carnosine dipeptidase 2	Cytosol, Nucleoplasm	Catalyzes the peptide bond hydrolysis in dipeptides.	3.7	13.6
**CTPS1**	A0A3B3IRI2; P17812; B4E1E0; A0A3B3IRQ8; A0A3B3ITF6; A0A3B3ITB8; A0A3B3ISY3; A0A3B3ITH9	CTP Synthase 1	Cytosol, in actin filaments	This enzyme is involved in the *de novo* synthesis of CTP, a precursor of DNA, RNA and phospholipids.	2.5	8.8
**DLD**	P09622; E9PEX6; F8WDM5; A0A1W2PR83; F2Z2E3	Dihydrolipoamide dehydrogenase	Mitochondria, Nucleoplasm	Lipoamide dehydrogenase is a component of the glycine cleavage system as well as an E3 component of three alpha-ketoacid dehydrogenase complexes.	10.0	15*
**DUT**	H0YNW5; P33316; A0A0C4DGL3; H0YKC5; H0YMM5; H0YKI0	Deoxyuridine Triphosphatase	Nucleoplasm	Catalyzes the cleavage of 2'-deoxyuridine 5'-triphosphate (dUTP) into 2'-deoxyuridine 5'-monophosphate (dUMP) and inorganic pyrophosphate and through its action efficiently prevents uracil misincorporation into DNA and at the same time provides dUMP, the substrate for *de novo* thymidylate biosynthesis.	2.2	6.7
**ECH1**	Q13011; M0R248; M0QZW4	Enoyl-CoA hydratase 1	Mitochondria	Isomerization of 3-trans,5-cis-dienoyl-CoA to 2-trans,4-trans-dienoyl-CoA.<br>**Fatty acid metabolism, Lipid metabolism**	6.0	15*
**GMDS**	O60547	GDP-mannose 4,6 dehydratase	Intracellular	Catalyzes the conversion of GDP-D-mannose to GDP-4-dehydro-6-deoxy-D-mannose.	3.6	10.0
**HADHA**	H0YFD6; P40939; A0A2R8Y4F5; A0A2R8YG21; A0A2R8Y688	Hydroxyacyl-CoA dehydrogenase trifunctional	Mitochondria	Mitochondrial trifunctional enzyme catalyzes the last three of the four reactions of the mitochondrial beta-oxidation pathway. HADH is a heterotetrameric complex composed of two proteins, the trifunctional	1.8	9.2

Gene name(s)	Protein Group IDs from MaxQuant MS analysis software	Protein name(s)	Localization	Protein function and biological process	Δ*Tm* / °C	-lg(adj. p, NPARC)
		multienzyme complex subunit alpha		enzyme subunit alpha/HADHA carries the 2,3-enoyl-CoA hydratase and the 3-hydroxyacyl-CoA dehydrogenase activities.<br>**Fatty acid metabolism, Lipid metabolism**		
**HADHB**	Q96KP4; J3QKT2; A0A087WYZ1; J3KSV5; J3QRD0; J3QL02; J3QRA8; J3QKQ0; J3QLU1; J3QR27; J3QQN6; A0A087WVS2; J3QRH4; J3KRD5; J3KSS4; J3KRJ8; J3QRP4	Hydroxyacyl-CoA dehydrogenase trifunctional multienzyme complex subunit beta	Mitochondria	See HADHA.<br>HADH is a heterotetrameric complex composed of two proteins, the trifunctional enzyme subunit beta/HADHB bears the 3-ketoacyl-CoA thiolase activity.<br>**Fatty acid metabolism, Lipid metabolism**	1.8	6.1
**ICAM1**	P05362; K7EKL8; E7ESS4	Intercellular adhesion molecule 1	Membrane	ICAM proteins are ligands for the leukocyte adhesion protein LFA-1 (integrin alpha-L/beta-2). During leukocyte trans-endothelial migration, ICAM1 engagement promotes the assembly of endothelial apical cups through ARHGEF26/SGEF and RHOG activation.	6.7	6.6
**IKBKG**	A0A087X0G7; A0A087WUW6; A0A087X1B1; Q9Y6K9; A0A087WV30; C9JN51; C9JH59; C9J2V2	Inhibitor of nuclear factor kappa B kinase regulatory subunit gamma	Cytosol, intracellular	Regulatory subunit of the IKK core complex which phosphorylates inhibitors of NF-kappa-B thus leading to the dissociation of the inhibitor/NF-kappa-B complex and ultimately the degradation of the inhibitor.	2.3	7.6
**MIEN1**	Q9BRT3 ;J3KTI2	Migration and invasion enhancer 1	Cytoplasm, cell membrane	Increases cell migration by inducing filopodia formation at the leading edge of migrating cells. Plays a role in regulation of apoptosis, possibly through control of CASP3. May be involved in a redox-related process.	6.5	6.6
**NLN**	A0A3B3IRI2; P17812; B4E1E0; A0A3B3IRQ8; A0A3B3ITF6; A0A3B3ITB8; A0A3B3ISY3; A0A3B3ITH9	Histidyl-tRNA synthetase 2, mitochondrial	Mitochondria	Mitochondrial aminoacyl-tRNA synthetase that catalyzes the ATP-dependent ligation of histidine to the 3'-end of its cognate tRNA, via the formation of an aminoacyl-adenylate intermediate (His-AMP).	1.4	6.1
**NUDT1**	H0YFD6; P40939; A0A2R8Y4F5; A0A2R8YG21; A0A2R8Y688	Peroxiredoxin 1	Mitochondria	Thiol-specific peroxidase that catalyzes the reduction of hydrogen peroxide and organic hydroperoxides to water and alcohols, respectively. Plays a role in cell protection against oxidative stress by detoxifying peroxides and as sensor of hydrogen peroxide-mediated signaling events.	1.4	6.1

Gene name(s)	Protein Group IDs from MaxQuant MS analysis software	Protein name(s)	Localization	Protein function and biological process	Δ*Tm* / °C	-lg(adj. p, NPARC)
**PITRM1**	Q5JRX3; A0A0A0MRX9; B1APQ0; H0Y4F7; H0Y7L7	Pitrilysin metallopeptidase 1	Mitochondria	Metalloendopeptidase of the mitochondrial matrix that functions in peptide cleavage and degradation, it degrades the transit peptides of mitochondrial proteins after their cleavage.	1.7	9.5
**PM20D2**	Q8IYS1	Peptidase M20 domain containing 2	Nucleoplasm	Catalyzes the peptide bond hydrolysis in dipeptides having basic amino acids lysine, ornithine or arginine at C-terminus. Postulated to function in a metabolite repair mechanism by eliminating alternate dipeptide by-products formed during carnosine synthesis.	4.9	15*
**RPS27A; UBC; UBB; UBA52**	Q9BYT8; E9PCB6; H0YAK4; H0YAF7	Ubiquitin-40S ribosomal protein S27a;Ubiquitin;40S ribosomal protein S27a;Polyubiquitin-C;Ubiquitin;Ubiquitin-60S ribosomal protein L40;Ubiquitin;60S ribosomal protein L40;Polyubiquitin-B;Ubiquitin	(Ubiquitin): Cytoplasm, nucleus. (Small ribosomal subunit protein eS31): Nucleus, nucleolus	(Ubiquitin): Exists either covalently attached to another protein, or free (unanchored). When covalently bound, it is conjugated to target proteins via an isopeptide bond either as a monomer (monoubiquitin), a polymer linked via different Lys residues of the ubiquitin (polyubiquitin chains) or a linear polymer linked via the initiator Met of the ubiquitin (linear polyubiquitin chains). Polyubiquitin chains, when attached to a target protein, have different functions depending on the Lys residue of the ubiquitin that is linked. (Small ribosomal subunit protein eS31): Component of the 40S subunit of the ribosome	3.6	6.1
**SDHB**	P21912; A0A087WWT1	Succinate dehydrogenase (ubiquinone) iron-sulfur subunit	Mitochondrion inner membrane	Iron-sulfur protein (IP) subunit of the succinate dehydrogenase complex (mitochondrial respiratory chain complex II), responsible for transferring electrons from succinate to ubiquinone (coenzyme Q).	1.7	6.5
**SUMO1**	B8ZZN6; P63165; B8ZZJ0; B9A032; G2XKQ0; B8ZZ67	Small ubiquitin like modifier 1	Nucleoplasm, Nuclear membrane, Nucleoli, Nuclear bodies	Ubiquitin-like protein that can be covalently attached to proteins as a monomer or a lysine-linked polymer. This post-translational modification on lysine residues of proteins plays a crucial role in a number of cellular processes such as nuclear transport, DNA replication and repair, mitosis and signal transduction. Involved for instance in targeting RANGAP1 to the nuclear pore complex protein RANBP2.	4.1	9.6
**TMED2**	Q15363; F5GX39; E7EQ72	Transmembrane emp24 domain-containing protein 2	ER, Golgi apparatus	Involved in vesicular protein trafficking. Mainly functions in the early secretory pathway but also in post-Golgi membranes.	1.9	9.4

Gene name(s)	Protein Group IDs from MaxQuant MS analysis software	Protein name(s)	Localization	Protein function and biological process	Δ*Tm* / °C	-lg(adj. p, NPARC)
**TMED9**	Q9BVK6	Transmembrane p24 trafficking protein 9	ER, Golgi apparatus	Involved in vesicular protein trafficking, mainly in the early secretory pathway.	1.8	9.7
**TMED10**	P49755; G3V2K7	Transmembrane p24 trafficking protein 10	Golgi apparatus	Cargo receptor involved in protein vesicular trafficking and quality control in the endoplasmic reticulum (ER) and Golgi.	1.3	6.2
**TRIP13**	Q15645; H0YAL2	Thyroid hormone receptor interactor 13	Nucleoplasm	Promotes early steps of the DNA double-strand breaks (DSBs) repair process upstream of the assembly of RAD51 complexes. Required for depletion of HORMAD1 and HORMAD2 from synapsed chromosomes (By similarity). Plays a role in mitotic spindle assembly checkpoint (SAC) activation.	2.7	11.5
**TUBA1B**	P68363; C9JDS9	Tubulin alpha 1 b	Microtubules	See TUBB.	5.7	15*
**TUBA1C**	F5H5D3; Q9BQE3; A0A1W2PQM2; F8VVB9; F8VRZ4; F8VS66; F8VWV9; F8VX09; F8VRK0;F8VXZ7;F8W0F6	Tubulin alpha 1 c	Microtubules	See TUBB and TUBA1B	4.4	9.3
**TUBB**	Q5JP53; P07437; Q5ST81; A6NNZ2; M0R1I1	Tubulin beta class I	Microtubules, Cytokinetic bridge, Mitotic spindle	Tubulin is the major constituent of microtubules. It binds two moles of GTP, one at an exchangeable site on the beta chain and one at a non-exchangeable site on the alpha chain.	6.9	15*
**TUBB4B; TUBB4A**	P68371; P04350; M0R2D3; M0QY85; M0QZL7; M0R278; M0R0X0; M0QY37; M0QX14; M0QYM7; M0R042; M0R2T4	Tubulin beta 4B class Ivb	Microtubules, Cytokinetic bridge, Mitotic spindle	See TUBB, TUBA1B, TUBA1C	3.7	12.7

*Zero value TPP software (R package) output for adjusted NPARC p-values; manually assigned with an arbitrary reasonable value of -lg(adj. p, NPARC) = 15 (the lowest non-zero adjusted NPARC p-value was 2.4×10^{-14}, i.e., -lg(adj. p, NPARC) = 13.6) after checking the correctness of data and melting curves.

SUPPLEMENTAL MATERIALS AND METHODS

Thermal proteome profiling - temperature range (TPP-TR)

Thermal proteome profiling with temperature range (TPP-TR) was essentially performed as described [3-6].

Compound and temperature treatment:

Ramos cells (Burkitt's B cell lymphoma) were expanded under standard cell culture conditions (37 °C, 5% CO_2, 100% rel. humidity) in culture medium (RPMI + 10 % FCS + 100 U/mL penicillin + 100 µg/mL streptomycin + 10 mM HEPES) to 75 million cells per replicate. Three replicates were performed on three consecutive days.

Per replicate, 1.87 million cells per mL in 20 mL culture medium were incubated with bromoxib (final concentration 40 µM; final 0.4% DMSO) for 30 min at 37°C. A vehicle control was performed side-by-side under the same conditions (same cell concentration and volume; final 0.4% DMSO). The cells were pelleted by centrifugation (1200 rpm, 5 min, 4°C), washed twice with ice cold PBS (39 mL and 1 mL) by resuspending, centrifugation, and aspiration of the supernatant, resuspended in 1 mL ice-cold PBS containing protease inhibitor cocktail (Roche mini, EDTA free) and split up into ten times 100 µL cell suspension in PCR tubes, respectively, such that each of the ten pairs of cell suspensions (treatment and control, 3.75 million cells, respectively) could be treated side-by-side at one of ten discrete temperatures, equally distributed in a range between 37°C and 68.5°C (7 min pre-incubation at rt., 3 min incubation at the discrete temperature, 3 min post-incubation at r.t. using pre-heated metal blocks for PCR tubes for uniform heat dissipation). After heat treatment, the samples were supplemented with Benzonase (final 0.4 U/mL) and 10x lysis buffer (final concentrations: 1.5 mM MgCl2, 1 mM Na_3VO_4, 10 mM NaF, 2.5 mM $Na_4P_2O_7$, 0.8% w/v NP-40), shock frozen in liquid nitrogen, and, after thawing, incubated on ice for 1 h. The crude lysates were centrifuged (20000 rcf, 30 min, 4 °C), the supernatants were recovered and the resulting cell extracts containing the fraction of soluble, non-denatured proteins were shock frozen in liquid nitrogen and stored at -80 °C after determining the total protein concentration (Pierce 660 nm Protein Assay, BSA as standard) of each of the two (treatment and control) times ten (temperatures) times three (replicates) = 60 samples. These samples were also used for quantitative immunoblotting (CETSA) as described below.

Single-pot, solid-phase-enhanced sample preparation (SP3):

SP3 was performed as described [5], using slight modifications to the original protocol [7]. The same initial volume was used for all 60 samples, thus, maintaining the information about the temperature dependent non-denatured protein content. This volume was calculated such that the lowest temperature samples (37 °C, three replicates) contained 10 µg total protein on average. The samples were diluted using SDS containing buffer (20 µL final, final concentrations: 7.5%

glycerol, 3% SDS, 37.5 mM Tris/HCl pH 7.0) and the proteins were reduced, alkylated, and precipitated on the solid phase as described [5] using adjusted volumes (2x). Volumes for washing of the aggregated proteins on the solid phase and for tryptic digestion were kept unchanged, however, to maintain the maximal total protein to trypsin/Lys-C ratio at 50:1 in the two rounds of digestion (13 h and 4 h), respectively, 2x 0.2 µg trypsin/Lys-C was used per sample, theoretically resulting in at most about 10 µg peptides in 22 µL 50 mM triethylammonium bicarbonate.

<u>TMT labeling and high pH fractionation:</u>

Peptides (10 µL of the peptide solutions, respectively, containing at most about 4.5 µg peptides, respectively) were TMT labeled (2 µL respective TMT 10plex label from 0.8 mg TMT label in 41 µL dry acetonitrile, 1 h, r.t.; quenched by 1.6 µL 2.5% w/v hydroxylamine, 15 min, r.t.), the samples of a labeling set were combined and offline high pH fractionated as described [5]. The ten different temperature treatments were split up into two TMT 10plex labeling sets as described [4] according to the following scheme:

TMT 10plex labeling set 1:

Temperature (°C)	65	65	58	58	51	51	44	44	37	37
Compound treatment	-	+	-	+	-	+	-	+	-	+
TMT label	126	127N	127C	128N	128C	129N	129C	130N	130C	131

TMT 10plex labeling set 2:

Temperature (°C)	68.5	68.5	61.5	61.5	54.5	54.5	47.5	47.5	40.5	40.5
Compound treatment	-	+	-	+	-	+	-	+	-	+
TMT label	126	127N	127C	128N	128C	129N	129C	130N	130C	131

<u>LC-MS/MS analysis:</u>

In total, eight (high pH fractions per TMT set) times two (TMT sets per replicate) times three (replicates) = 48 samples were analyzed using a Rapid Separation Liquid Chromatography System (Ultimate 3000, Thermo Fisher) and a nano-source ESI interface equipped Orbitrap Fusion Lumos Tribrid mass spectrometer (Thermo Fisher Scientific, Dreieich, Germany) operated in synchronous precursor selection (SPS [8]) mode as described [5].

<u>MS data analysis, protein identification, and quantification:</u>

MS data was processed as described [5] using the MaxQuant software (Max Planck Institute for Biochemistry, Planegg, Germany) version 2.0.3.0 based on 75777 Homo sapiens protein entries, downloaded from the UniProtKB on 27 January 2021, yielding protein quantifications by TMT

reporter ions at the MS3 level for a total of 4293 identified protein groups (including potential contaminants, reverse hits and only by site identifications). In the following, for simplicity and readability, a MaxQuant "protein group" is referred to as "identified protein", "protein ID", or just "protein", and a representative protein for the protein group is selected.

Statistical analysis of melting curves:

Statistical data analysis was performed using the R programming language (R versions 4.1.2 and 4.3.2 on a x86_64-w64-mingw32/x64 (64-bit) platform) based in principle on ten (reporter ion intensities per TMT set) times two (TMT sets per replicate) times three (replicates) = 60 reporter ion intensity values per protein as output of the MaxQuant software. In practice, missing reporter ion intensity values can occur i) in the absence of protein identification in one or more TMT sets, resulting in missing values for the entire TMT set(s), or ii) when the reporter ion intensity falls below a certain threshold (more likely at elevated temperatures). In the present case, one sample (37 °C, control, replicate two) was excluded from the analysis because it appeared as outlier in principle component and cluster analyses performed as quality control.

The melting curve analysis procedure consisted of two main steps: 1) Preparation of TPP-TR data for subsequent fitting, 2) fitting of melting curves and statistical analysis using the TPP R package.

1) TPP-TR data were prepared from the TMT reporter ion intensities by a three-step normalization procedure (i) “Normalization for each temperature”, ii) “Normalization to a global melting curve”, and iii) “Normalization of protein-wise melting curves from each TMT 10plex labeling set”) essentially as described [4] using the nls() or nlsLM() functions of the R packages stats or minpack.lm, respectively, for non-linear melting curve fitting (R version 4.1.2). Only proteins with at least 20 valid reporter ion intensity values were considered (3973 proteins). For step iii), to increase the robustness of fit, four (comprising all combinations of I_{min} = 0 or "minimum protein intensity" and I_{max} = "mean protein intensity at the lowest temperature" or "maximum protein intensity" as fixed parameters) two-parameter fits were first performed using the nlsLM() function to obtain rough estimates of the melting point (T_m, initial value 52.75 °C) and slope (s, initial value -0.5) parameters for each protein. The two-parameter fit with the lowest residual standard error (σ) was selected and its parameters (I_{min}, I_{max}, T_m, and s) were chosen as initial values for the subsequent four-parameter fit.

2) Fitting of melting curves

2.1) First, as described [4] for the third normalization step (iii), melting curves for each protein were fitted to the combined data of all six labeling sets using two and then four parameter (I_{min}, I_{max}, T_m, and s) fits.

2.2) The parameters of the latest (four parameter) melting curve fits from 2.1) were used as constants (I_{min}, I_{max}) or initial values (T_m, s) for the next round of melting curve fits per protein, which employed scaling factors for differential protein expression $sf_{t,c}$ and the melting curve parameters $T_{m\text{-}t,c}$ and $s_{t,c}$ as fitting parameters separately for treatment (suffix t) and control (suffix c) data (Eq. 1). The incorporation of stacked variables (with v_t as a factor taking the value 1 or 0 for data from treatment and control, respectively, and v_c as a factor taking the value 1 or 0 for data from control and treatment, respectively) allowed the more stable nlsLM() algorithm to be used instead of nls(), resulting in fewer errors in the fitting algorithm.

$$I(T) = (v_t\, sf_t + v_c\, sf_c)\ (\ I_{min} + (I_{max} - I_{min})\ /\ (1 + \exp[((v_t\, T_{m\text{-}t} + v_c\, T_{m\text{-}c})/T - 1)\ (\ v_t\, s_t + v_c\, s_c)\ (\ v_t\, T_{m\text{-}t} + v_c\, T_{m\text{-}c})])\ ) \qquad \text{(Eq. 1)}$$

For each, treatment and control, the number of data points for the melting curve fit was five (temperatures per labeling set) times two (labeling sets) times three (replicates) = 30 (29 for the control due to the removed outlier, see above), i.e., three data points per temperature (two for 37°C of the control due to the removed outlier of replicate two), for a complete set of valid values of reporter ion intensities.

2.3) Melting curves were normalized to a maximum plateau (I_{max}) of 1 using the fitted parameters I_{max} (step 2.1) and $sf_{t,c}$ (step 2.2) separately for treatment and control.

2.4) To determine effects of compound treatment on protein thermal stability, statistical analysis of melting curves was performed essentially as described [6] using the TPP R package version 3.30.0 [9] in R version 4.3.2 without further internal normalization (parameter "normalize = F") and comparing all three treatment replicates with all three control replicates. For each protein, the null hypothesis (same melting for treatment and control) was tested and an adjusted p-value was calculated by the NPARC algorithm within the TPP package. The negative decadic logarithm of this adjusted NPARC p-value, -lg(adj. p, NPARC), was plotted against the difference of the arithmetic means of the melting points of treatment and controls, ΔT_m (Figure 7A in the main article). Proteins with an incomplete set of valid reporter ion intensity values (except for the removed outlier, see above), potential contaminants, reverse hits and "by site" identifications were omitted as well as proteins identified with only 1 Razor & unique peptide or that had $R^2 \leq 0.8$ for one or more melting curve fits, resulting in 1985 remaining proteins. If proteins had zero value TPP software outputs for their adjusted NPARC p-values, they were manually assigned with an arbitrary reasonable value of -lg(adj. p, NPARC) = 15 (the lowest non-zero adjusted NPARC p-value output was 2.4×10^{-14}, i.e., -lg(adj. p, NPARC) = 13.6) after checking the correctness of data and melting curves. Proteins with -lg(adj. p, NPARC) > 6 and $\Delta T_m > 0.5$ were regarded as significantly stabilized (31 proteins) by direct or indirect effects of bromoxib treatment and were further analyzed for protein-protein association networks using the online STRING data base online tool (https://string-db.org, version 11.5; [10]).

General remarks:

For all melting curve fits using the nls() or nlsLM() functions in R, the melting point fitting parameter (T_m) was constrained between the lowest and highest treatment temperatures (37-68.5 °C) and the minimum and maximum intensity fitting parameters (I_{min} and I_{max}) were constrained between 0 and 110% of the maximum intensity value of the data used for fitting; likewise, scaling factors (*sf*) were constraint between 0 and 110% of the ratio between the maximum intensity data value and I_{max} (constant from previous fit without scaling factors). Additionally, a weighting of datapoints was applied as the respective ratio between each originally determined protein reporter intensity (MaxQuant output) and the (normalized) intensity actually used for the fit to reduce the influence of upscaled data with initially relatively low intensity (exhibiting lower quality due to, e.g., higher relative variance when compared to higher intensity data).

Cellular thermal shift assay (CETSA)

Validation of bromoxib mediated thermal stabilization of ECH1, ACADVL, ACSL4, and HADHB by quantitative immunoblotting (CETSA [11]) was essentially performed as described [4].

For CETSA analysis, the TPP-TR cell extract samples (see above) were employed and the same initial volume was used for all 54 samples (nine temperatures equally distributed between 41.5 °C and 68.5 °C, treatment and control, three replicates), thus, maintaining the information about the temperature dependent non-denatured protein content. This volume was calculated from the six lowest temperature samples (37 °C, treatment and control, three replicates) to contain 20 µg total protein on average. The samples were supplemented with sample buffer (final concentrations: 62.5 mM Tris, 8.6% [v/v] glycerol, 2% [w/v] SDS, 33.3 µg/mL bromophenol blue, 1% [v/v] β-mercaptoethanol) and heated at 95 °C for 5 min before loading on SDS-polyacrylamide gels. After separation by SDS-PAGE, proteins were transferred to PVDF membranes (Merck, Darmstadt, Germany, #IPFL00010), blocked with 5% milk powder in TBST and analyzed using the indicated primary antibodies followed by appropriate IRDye 800- or IRDye 680-conjugated secondary antibodies (LI-COR Biosciences, Lincoln, NE, USA). Fluorescence signals were detected using an Odyssey Infrared Imaging system (LI-COR Biosciences, Lincoln, NE, USA) and signals were quantified with Image Studio (LI-COR Biosciences, Lincoln, NE, USA). For CETSA protein melting representations, normalized quantitative immunoblot data was used. Normalization was performed by dividing each of the two (treatment and control) x three (replicates) = six data sets (nine temperatures each) per protein by the respective fitted I_{max} (constrained to at most 110% of the maximum intensity value of the data used for fitting) of three parameter (I_{max}, T_m, and *s*; I_{min} = 0) melting curve fits [4].

Supplemental References

1. Uniprot. Uniprot updated: Release 2023_01, Access Date 22.03.2023 [Available from: https://www.uniprot.org/.
2. THPA. The human protein atlas Version: 22.0, Atlas updated: 2022-12-07, Access Date 22.03.2023 [Available from: https://www.proteinatlas.org/.
3. Franken H, Mathieson T, Childs D, Sweetman GM, Werner T, Tögel I, et al. Thermal proteome profiling for unbiased identification of direct and indirect drug targets using multiplexed quantitative mass spectrometry. Nat Protoc. 2015;10:1567-93.
4. Berning L, Lenz T, Bergmann AK, Poschmann G, Brass HU, Schlütermann D, et al. The Golgi stacking protein GRASP55 is targeted by the natural compound prodigiosin. Cell Commun Signal. 2023;21:275.
5. Lenz T, Stühler K. Small Molecule Arranged Thermal Proximity Coaggregation (smarTPCA) - A Novel Approach to Characterize Protein-Protein Interactions in Living Cells by Similar Isothermal Dose-Responses. Int J Mol Sci. 2022;23:5605.
6. Stuhldreier F, Schmitt L, Lenz T, Hinxlage I, Zimmermann M, Wollnitzke P, et al. The mycotoxin viriditoxin induces leukemia- and lymphoma-specific apoptosis by targeting mitochondrial metabolism. Cell Death Dis. 2022;13:938.
7. Hughes CS, Moggridge S, Muller T, Sorensen PH, Morin GB, Krijgsveld J. Single-pot, solid-phase-enhanced sample preparation for proteomics experiments. Nat Protoc. 2019;14:68-85.
8. McAlister GC, Nusinow DP, Jedrychowski MP, Wühr M, Huttlin EL, Erickson BK, et al. MultiNotch MS3 Enables Accurate, Sensitive, and Multiplexed Detection of Differential Expression across Cancer Cell Line Proteomes. Anal Chem. 2014;86:7150-8.
9. Childs D, Kurzawa N, Franken H, Doce C, Savitski M, Huber W. TPP: Analyze thermal proteome profiling (TPP) experiments. doi:10.18129/B9.bioc.TPP, R package version 3.30.0, https://bioconductor.org/packages/TPP. R package version. 2023;3:0.
10. Szklarczyk D, Gable AL, Lyon D, Junge A, Wyder S, Huerta-Cepas J, et al. STRING v11: protein–protein association networks with increased coverage, supporting functional discovery in genome-wide experimental datasets. Nucleic Acids Res. 2019;47:D607-D13.
11. Jafari R, Almqvist H, Axelsson H, Ignatushchenko M, Lundbäck T, Nordlund P, et al. The cellular thermal shift assay for evaluating drug target interactions in cells. Nat Protoc. 2014;9:2100-22.
